# Region-dependent mechanical parameters in simulating cerebral atrophy

**DOI:** 10.1063/5.0294034

**Published:** 2026-01-08

**Authors:** Nicole Tueni, Emma Griffiths, Johannes Weickenmeier, Stefan Rampp, Silvia Budday

**Affiliations:** 1 Institute of Continuum Mechanics and Biomechanics, Department of Mechanical Engineering, Friedrich-Alexander-Universität Erlangen-Nürnberg, 91058 Erlangen, Germany; 2 Department of Engineering Science, University of Oxford, Oxford OX3 7DQ, United Kingdom; 3 Departments of Neurosurgery & Neuroradiology, Universitätsklinikum Erlangen, Friedrich-Alexander-Universität Erlangen-Nürnberg, Erlangen 91054, Germany

## Abstract

Brain aging and atrophy involve complex multiscale factors of cellular degeneration and morphological changes. Although previous biomechanical models have advanced our understanding of brain shrinkage due to physiological and pathological aging, they often rely on simplified representations of tissue properties with limited regional differentiation. Building on established links between regional mechanics and neurodegeneration, we extend atrophy models by introducing detailed mechanical heterogeneity across 17 anatomically and mechanically distinct brain regions. Using region-specific experimental material properties, the model differentiates local mechanical behavior and reveals effects that homogeneous models overlook, providing new clinical insight into region-specific vulnerabilities. We then compare this heterogeneous model with progressively simplified variants to assess the impact of regional variability on brain deformation. While global and regional volume changes remain largely unaffected by mechanical heterogeneity, volume fractions of the corpus callosum and ventricles are sensitive to regional differences in material parameters. Analysis of the displacement field shows that mechanical heterogeneity significantly influences local displacement patterns within brain regions. Stress and stretch analyses reveal discrepancies between simplified and heterogeneous models, particularly in the corpus callosum, the internal brain structures, and some cortical regions. These results emphasize the importance of incorporating regional mechanical heterogeneity to enhance the accuracy of brain simulations and underscore the need for more comprehensive experimental characterization of brain tissue properties.

## I. INTRODUCTION

Brain aging is a complex process that encompasses alterations at multiple levels, ranging from the cellular to the macroscopic scales. At the cellular level, aging is characterized by a decrease in neuronal and synaptic function.^1^ At the vascular level, aging-related changes in cerebral vasculature lead to reduced cerebral blood flow, contributing to neurodegenerative processes.^2^ On the macroscopic scale, morphological changes manifest as progressive loss of brain tissue, resulting in volume reduction, ventricular enlargement, and cortical thinning.^3–5^ These processes can occur physiologically with aging or pathologically due to neurodegenerative diseases, such as Alzheimer's disease and dementia.^6,7^

Extensive research has been conducted from biological and medical perspectives, showing that neurotoxic protein accumulation plays a central role in brain aging and neurodegenerative diseases. In Alzheimer's disease, two proteins are particularly significant: *Amyloid* β, which forms extracellular plaques that disrupt neuronal communication, and *Tau*, which creates intracellular tangles that destabilize neuronal structure.^8^

The literature shows that different brain regions exhibit selective atrophy in neurodegenerative diseases and that mechanical differences could contribute to these selective changes. Aging alters global brain mechanics,^9^ and mechanical properties relate to tissue health and microstructure.^10^ Moreover, more substantial softening has been reported in the frontal, parietal, and temporal lobes in Alzheimer's disease,^11^ and medial temporal lobe stiffness has been linked to faster cognitive decline, suggesting that biomechanical measures may provide valuable prognostic information.^12^

From a biomechanical perspective, pioneering models have been developed to shed more light on brain aging: Castellano-Smith *et al.*^13^ developed a phenomenological model of brain atrophy within a finite element framework, which includes morphological changes in the tissue. Harris *et al.*^14^ introduced a two-dimensional (2D) brain model to simulate volume loss after traumatic brain injury using a quasi-incompressible and hyper-elastic neo-Hookean material, distinguishing between gray matter (GM) and white matter (WM) atrophy. Weickenmeier *et al.*^15^ further developed a transport model simulating the anisotropic diffusion of neurotoxic proteins in the brain, accounting for differential shrinkage of GM and WM based on protein concentration. In their subsequent work, Weickenmeier *et al.*^16^ extended their model to Alzheimer's disease, Parkinson's disease, and amyotrophic lateral sclerosis (ALS). Schäfer *et al.*^17^ developed a multiphysics model that couples misfolded *Tau* protein spreading with tissue atrophy, using a neo-Hookean WM–GM material model, with greater GM shrinkage. Budday *et al.*^18^ applied a framework similar to brain growth models, using a multiplicative decomposition of the deformation gradient to simulate brain volume loss, considering a linear decrease in brain volume, by assuming a rate parameter dependent on the toxic protein concentration. Building on these prior works, Blinkouskaya *et al.*^19^ applied Schäfer's model with regional isotropic shrinkage in a 3D brain model, incorporating a neo-Hookean material with distinct WM and GM mechanical properties and a differentiation of atrophy rates between WM, GM, and the hippocampus.

Although these studies have advanced our understanding of brain atrophy, they generally rely on brain models with limited regional differentiation. The human brain consists of several anatomical regions, each with distinct microstructural architecture.^20,21^ These microstructural differences result in variations in mechanical behavior,^22–26^ which underscore the importance of incorporating regional mechanical heterogeneity in computational models of brain atrophy. Previous studies^27,28^ have investigated the role of region-specific material properties in full-scale brain simulations under arbitrary loadings by segmenting the brain into nine distinct regions and incorporating experimentally derived mechanical properties.^29^ Comparisons with models of decreasing heterogeneity revealed that incorporating regional mechanical differences leads to significant variations in predicted stretch distributions compared to models assuming uniform material properties.

This study builds on previous research by extending the approach of incorporating regional mechanical heterogeneity into brain atrophy models. While previous studies have segmented the brain into nine regions,^27,28^ we propose a more detailed model by increasing the number of regions from nine to 17. This segmentation provides a more accurate representation of the brain, accounting for finer regional variations in mechanical behavior and enabling more precise predictions of stretch distributions within the brain.

## II. RESULTS AND DISCUSSION

Atrophy simulations were performed on six generated 3D brain models to evaluate (i) the effect of brain regional heterogeneity on structural and mechanical responses and (ii) the impact of atrophy progression on cerebral morphological changes using the most refined 17 Region (17R) model. The use of multiple brain models ensured the consistency of results and confirmed that the observed trends were not dependent on a single brain geometry.

### A. Effect of regional heterogeneity

The effect of regional heterogeneity in the brain on full-scale atrophy simulations was investigated by comparing the fully heterogeneous model (17R) with four models of decreasing heterogeneity (9R, 4R, 2R, and 1R).

#### 1. Morphology

To evaluate the influence of regional heterogeneity on morphological alterations during atrophy progression, volumetric changes were evaluated in the 3D brains for each regional model. A consistent reduction in total brain volume was observed as atrophy advanced, for all regional models.

The regional change in the volume fraction was quantified as the difference between the initial volume fraction (before the onset of atrophy) and the final volume fraction at the end of the simulation. For most regions, the effect of model heterogeneity on volume change was negligible, as demonstrated in Fig. 17 (Appendix B). Consistent with clinical signs of cerebral atrophy, the cerebrospinal fluid (CSF) layer showed a clear increase in volume, as illustrated in Fig. 16.

**FIG. 1.**
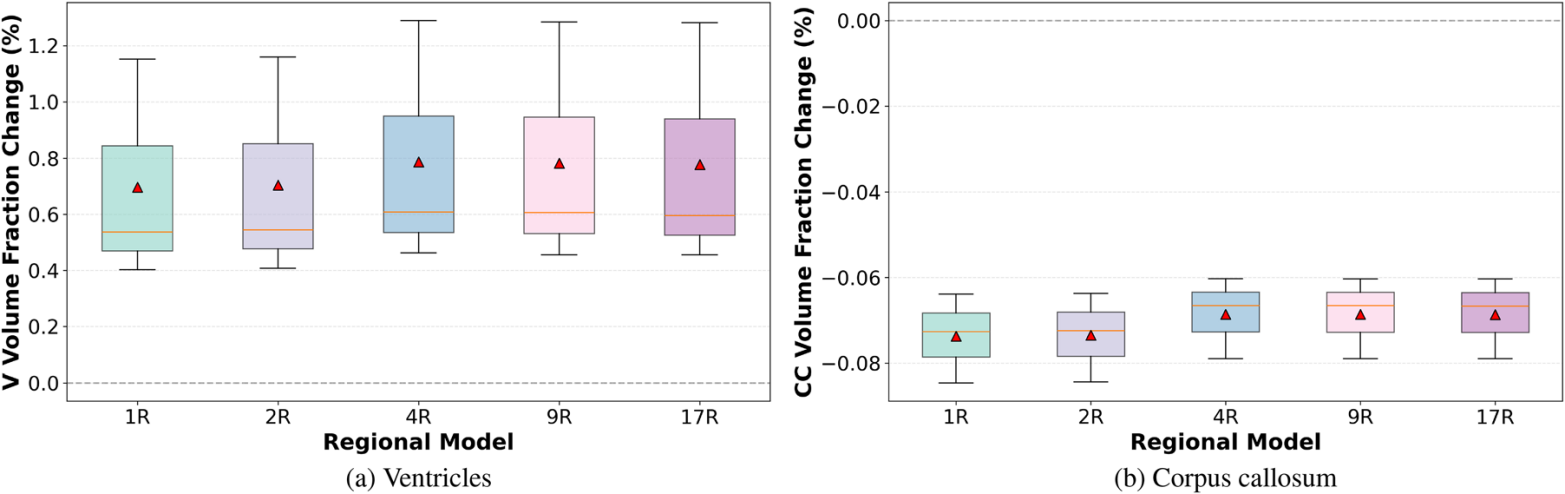
Boxplots showing the change in volume fraction across the simulated brains for each regional segmentation for (a) ventricles (V) and (b) corpus callosum (CC). Volume fraction change was quantified as the difference between the initial volume fraction (prior to atrophy) and the final volume fraction.

However, certain regions showed sensitivity to regional model configuration. As shown in Fig. 1(a), the ventricles showed an increase in volume fraction, indicating ventricular enlargement. This increase was more pronounced in the 4R, 9R, and 17R models compared to the 1R and 2R ones. In contrast, Fig. 1(b) shows a reduction in volume fraction for the corpus callosum, with a greater shrinkage observed in the homogeneous regional models (1R and 2R), compared to the more heterogeneous ones.

It is important to note that the reported volume changes are based on a simplified model in which the concentration of misfolded neurotoxic protein *c* is constant. This approach does not capture the accumulation and spread of the protein over time, as considered in more detailed models such as.^19^ As a result, the volume changes may not fully capture the complexity of atrophy progression. Nevertheless, this analysis was designed to highlight the effects of brain segmentation and regional mechanical properties on overall morphological changes.

#### 2. Displacements

**FIG. 2.**
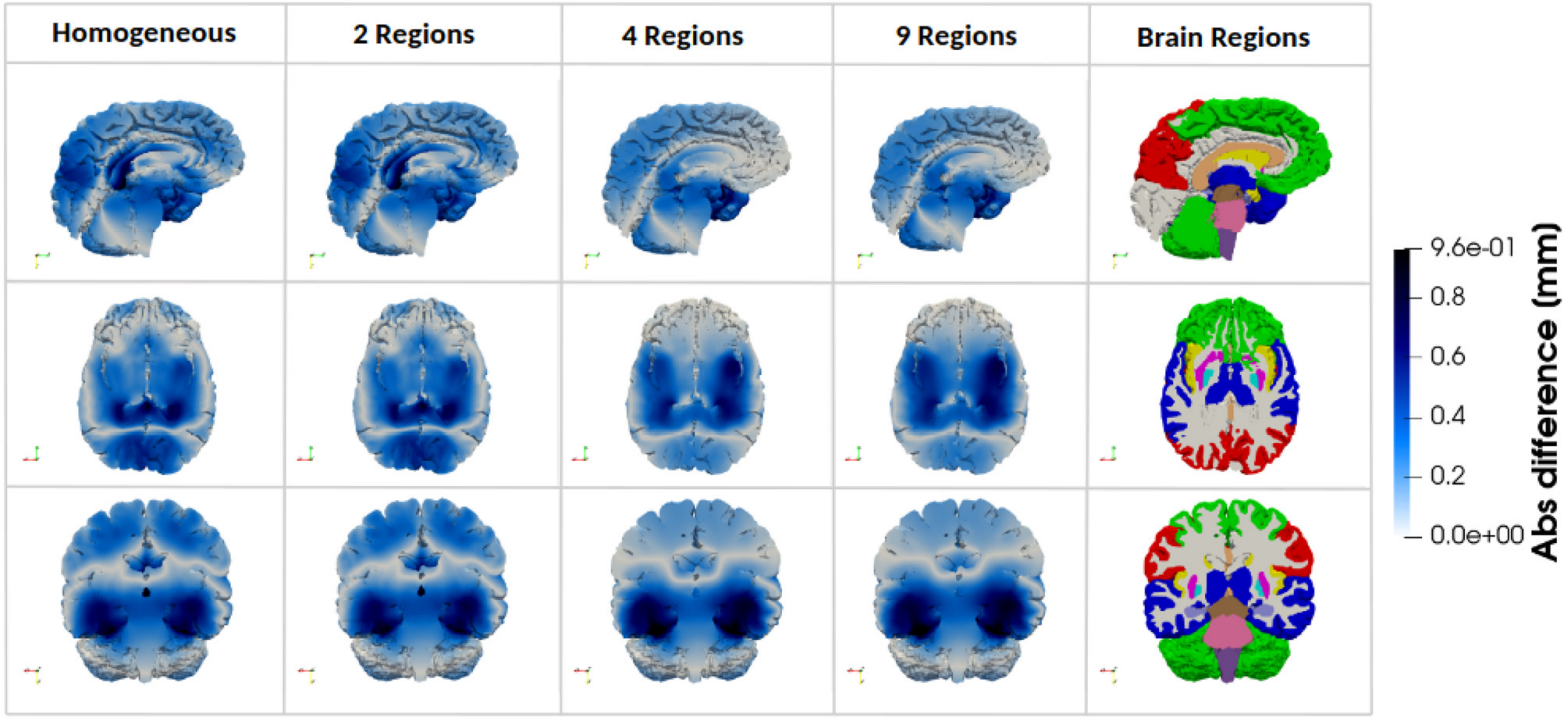
Absolute difference in simulated displacement field between the fully heterogeneous model (17 Regions) and the four simplified models (Homogeneous material properties, 2 Regions, 4 Regions, and 9 Regions) in three anatomical planes, for one representative 3D brain model.

To assess the effect of regional mechanical heterogeneity on brain kinematics, we first analyze the displacement field across the different brain regions. Figure 2 displays the absolute differences in the simulated displacement fields between the fully heterogeneous 17R model and the simplified models (1R, 2R, 4R, and 9R) in three anatomical planes. The sagittal plane shows that increasing heterogeneity reduces displacement differences in the motor cortex, frontal cortex, visual cortex, and corpus callosum. In contrast, the transverse plane reveals increased differences in the pallidum, putamen, hippocampus, and amygdala. In the coronal plane, simplified models show large differences in cortical areas that progressively diminish with greater heterogeneity.

In the corona radiata specifically, differences in displacements between the 17R model and progressively more homogeneous models do not vary linearly with heterogeneity. While displacement differences decrease in the sagittal and coronal planes as heterogeneity increases, they increase in the transverse plane. This discrepancy occurs because each anatomical plane captures different parts within the region.

**FIG. 3.**
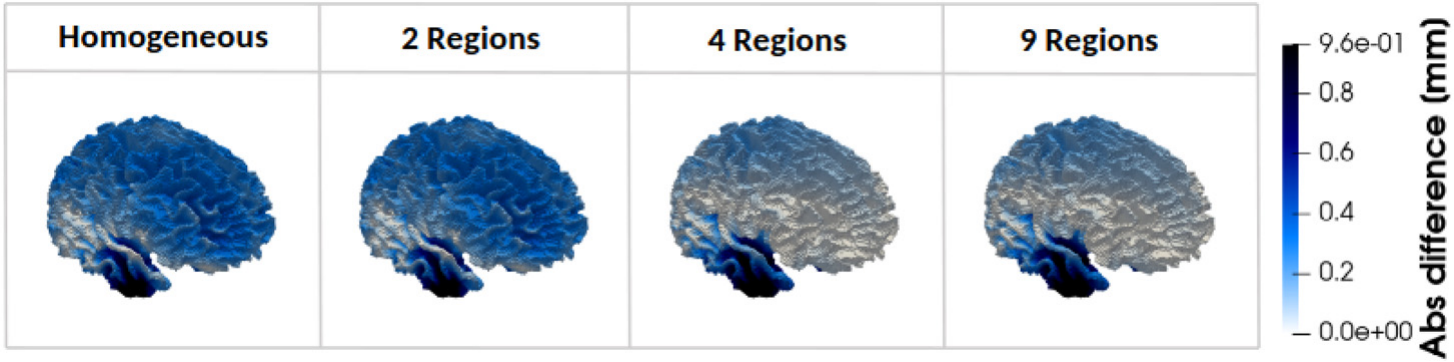
Absolute difference in simulated displacement field between the fully heterogeneous model (17 Regions) and the four simplified models (homogeneous material properties, 2 Regions, 4 Regions, and 9 Regions) within the Corona Radiata.

To investigate this variation in more detail, Fig. 3 shows the evolution of absolute differences in the simulated displacement within the corona radiata. The 1R and 2R models yield similar displacement distributions in this region, with a marked reduction in differences upon introducing the 4R model. Regionally, as heterogeneity increases, differences decrease near the frontal and motor cortex but increase toward the brainstem and cerebellum.

**FIG. 4.**
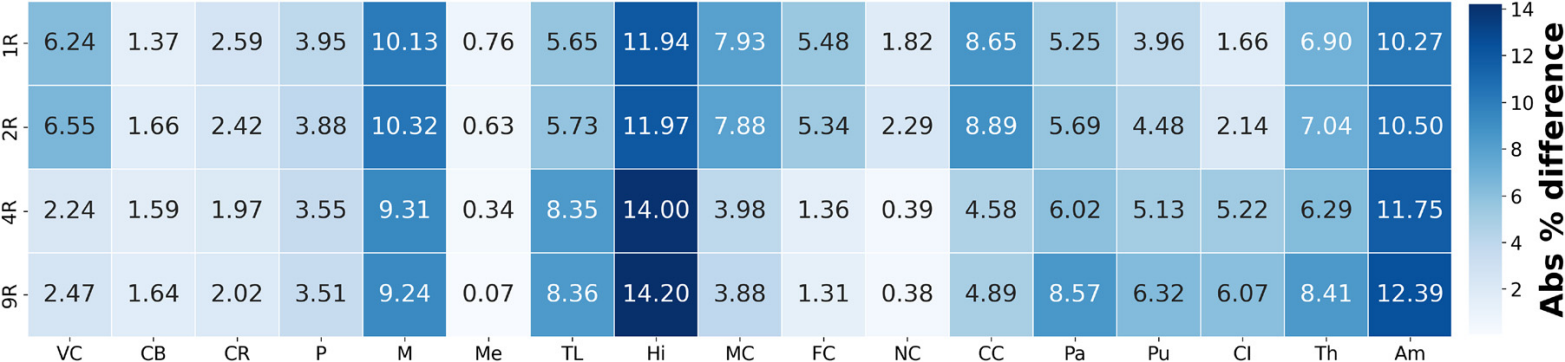
Heatmap of absolute percentage difference in average displacement field in each region between the fully heterogeneous model (17 Regions) and the four simplified regional models (homogeneous material properties, 2 Regions, 4 Regions, and 9 Regions).

Figure 4 presents a heatmap of the average absolute differences in displacement magnitude between the 17R model and the more homogeneous models. Differences generally decrease with increasing heterogeneity in cortical regions and the corpus callosum; increase in inner structures such as the basal ganglia, amygdala, and hippocampus; and remain relatively constant in the corona radiata, brainstem, and cerebellum, indicating limited sensitivity to heterogeneity in these areas.

Hu *et al.*^44^ investigated the characteristics of atrophy, revealing that the largest changes occur in the hippocampus, amygdala, temporal lobe, frontal/insular cortex, thalamus, basal ganglia, corpus callosum, and corona radiata. In our model, the displacement field agrees with these patterns, showing large displacement values in the brainstem, cerebellum, cortex, corona radiata, basal ganglia, hippocampus, and corpus callosum. Moreover, the hippocampus, amygdala, temporal lobe, midbrain, thalamus, and pallidum show high sensitivity in our analysis with important differences remaining between the 17R and 9R models. These differences indicate the importance of regional differentiation in finite element models of brain atrophy.

#### 3. Stresses and principal stretches

To further study the impact of region-dependent mechanical parameters in simulating cerebral atrophy, other mechanical outputs from the simulations, specifically stresses and stretches, reveal differences between the regional models. This section focuses on hydrostatic stress and the third principal stretch.

All other mechanical outputs, including von Mises stresses, first and second principal stretches, and maximum shear, are provided in Appendix B. Figures 18, 20, 22, and 24 illustrate the simulated first and second principal stretches, maximum shear, and von Mises stresses in three anatomical planes for brain models with increasing regional heterogeneity, whereas Figs. 19, 21, 23, and 25 depict the absolute differences in these quantities between the fully heterogeneous model and the four simplified models. Representative boxplots comparing the distributions of these quantities between the 9R and 17R models across all brain regions are shown in Figs. 28, 29, 30, and 31. Additionally, Fig. 32 presents the corresponding boxplots for hydrostatic stress.

**FIG. 5.**
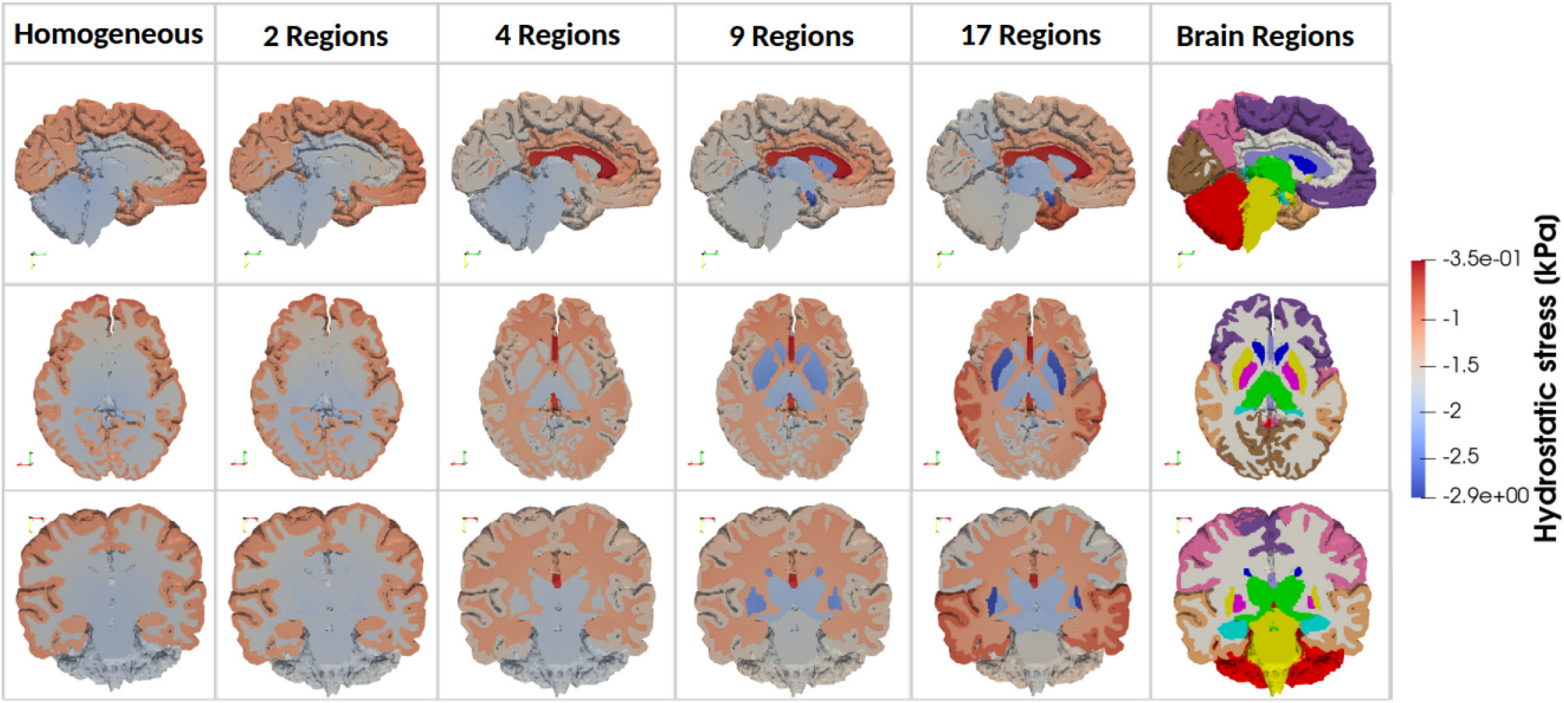
Simulated hydrostatic stress in three anatomical planes for one representative brain model with increasing regional heterogeneity from left to right (homogeneous, 2 Regions, 4 Regions, 9 Regions, and 17 Regions). Considered brain regions are indicated on the right.

*Hydrostatic stress*. Figure 5 shows the distribution of hydrostatic stress within one representative brain for all regional models. In all models, the hydrostatic stress remained consistently negative, indicating compressive loading conditions.

As the number of regions increases, differences in hydrostatic stress between regions become more pronounced. This occurs because the hydrostatic stress represents the pressure component of the stress tensor, which is linearly governed by the material parameters μ, α, and κ. The hydrostatic stress appears approximately constant within each region due to the use of a single set of material parameters per region. Since μ, α, and κ remain constant within each region, the resulting stress response is homogeneous throughout that region, creating the characteristic flat stress distribution observed within regions while maintaining sharp transitions at region interfaces.

The 1R and 2R models exhibit nearly identical patterns, with a relatively uniform hydrostatic stress, suggesting that a coarse regional differentiation in material properties has little impact on the overall stress distribution. However, in the 4R model, significant changes in hydrostatic stress emerge in certain brain regions, particularly in the cortex, where an increase is observed due to its greater stiffness compared to the subcortical regions. In contrast, a decrease appears in the corona radiata and corpus callosum, highlighting the contrast between the stiff cortex and the softer corona radiata. The 9R model introduces further differences, with an increase in hydrostatic stress in internal brain structures, primarily the midbrain and brainstem. Finally, in the 17R model, additional regional variations are observed. Specifically, hydrostatic stress increases in the motor cortex, while a decrease appears in the temporal lobe and cortex insula.

It should be emphasized that these results are purely mechanical, and their clinical or physiological relevance has yet to be established. They are presented in this study in order to highlight the effects of regional mechanical properties in the brain on stress distributions.

Here, too, the regions identified by Ref. 44 as highly sensitive to atrophy are of utmost relevance. Mechanical stress concentrations in such regions could make them more vulnerable to structural damage, potentially accelerating local atrophy. Our model shows that the highest stresses are observed in the corpus callosum, temporal lobe, frontal cortex, insular cortex (CI), and corona radiata; regions also sensitive to cerebral atrophy.

**FIG. 6.**
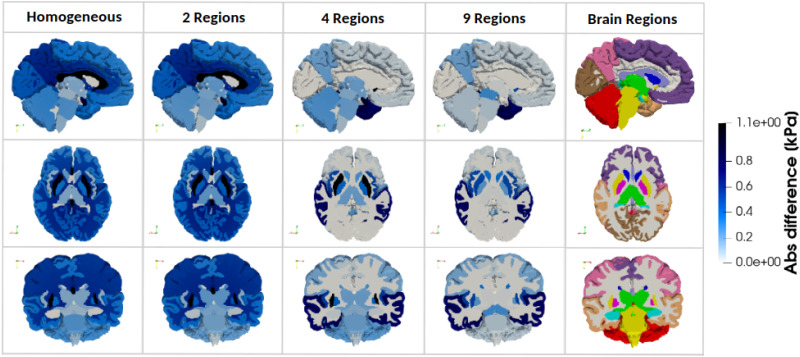
Absolute difference in hydrostatic pressure between the fully heterogeneous model (17 Regions) and the four simplified models (homogeneous material properties, 2 Regions, 4 Regions, and 9 Regions) in three anatomical planes, for one representative 3D brain model.

Figure 6 illustrates the distribution of the absolute difference in hydrostatic stress between the most refined, 17R model and the models with decreasing heterogeneity, for one representative brain. The homogeneous and 2R models exhibit significant discrepancies compared to the 17R model, particularly in the corpus callosum, internal brain structures, and corona radiata. This reflects the oversimplified material representation in the 1R and 2R models, suggesting that these assumptions may not be suitable for accurate brain simulations.

As observed in Fig. 5, the 4R model exhibits an important progression compared to the 1R and 2R models, particularly in the cortex, corona radiata, and corpus callosum, bringing the 4R model closer in behavior to the 17R model. However, as depicted in Fig. 6, significant differences persist between the 4R and 17R models, especially in the internal brain structures, the temporal lobe, and the insular cortex.

Compared to the 4R model, the 9R model more closely resembles the 17R model, with reduced regional differences in predicted hydrostatic stresses, particularly within the internal brain structures. However, significant differences remain in the temporal lobe and insular cortex, as depicted in the three anatomical planes shown in Fig. 6. These findings are consistent with the displacement results: sensitivity to regional heterogeneity is likewise most pronounced in regions known to be vulnerable to atrophy, further underscoring the importance of incorporating regional heterogeneity in brain mechanical modeling.

**FIG. 7.**
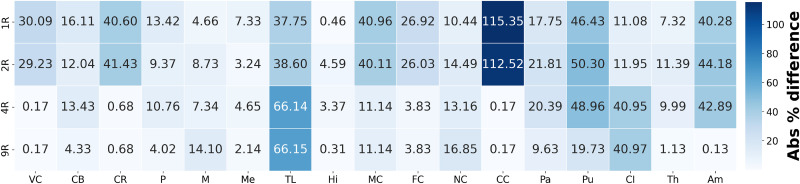
Heatmap of absolute percentage difference in average hydrostatic stress in each region (averaged over all 3D brain meshes) between the fully heterogeneous model (17 Regions) and the four simplified regional models (homogeneous material properties, 2 Regions, 4 Regions, and 9 Regions).

Figure 7 shows a heatmap of the absolute difference in average hydrostatic stress for each region, comparing the fully heterogeneous model (17R) with the other four models (averaged over all 3D brain meshes). Results are obtained by computing the absolute percentage differences for each individual brain mesh, then averaging these differences across all brains for each brain region and regional model. This visualization reinforces the observations from Fig. 6: in general, increasing model heterogeneity reduces discrepancies in predicted stresses compared to the fully heterogeneous model. Specifically, the differences gradually decrease in the cortex, corpus callosum, and corona radiata starting from the 4R model, and in the internal brain structures starting from the 9R model.

Large jumps in hydrostatic stress differences are observed between the 1R, 2R, and the 4R models. These are primarily due to the introduction of region-specific material parameters in the corona radiata, internal brain structures, and corpus callosum, which significantly alter the stress distributions across different brain regions. In the cortical regions (VC, MC, FC), the 4R model shows a decrease in difference compared to the more homogeneous models, due to its greater stiffness relative to the subcortical regions. The corpus callosum also exhibits a decrease in difference, as it is assigned a distinct set of material properties in the 4R model, whereas in the 1R and 2R models, it was grouped with the subcortical region. Similar patterns are observed in the transition from the 4R to the 9R model, due to the introduction of additional region-specific material parameters that further alter the stress distributions throughout the brain. The most significant changes occur in the internal brain structures, which are further segmented in the 9R model into distinct regions, including the basal ganglia, brain stem, midbrain, cerebellum, amygdala, and hippocampus, each with its own material properties. Finally, notable differences between the 17R and 9R models persist in the temporal lobe and insular cortex, highlighting the added value of using an even more refined model than the 9R configuration.

**FIG. 8.**
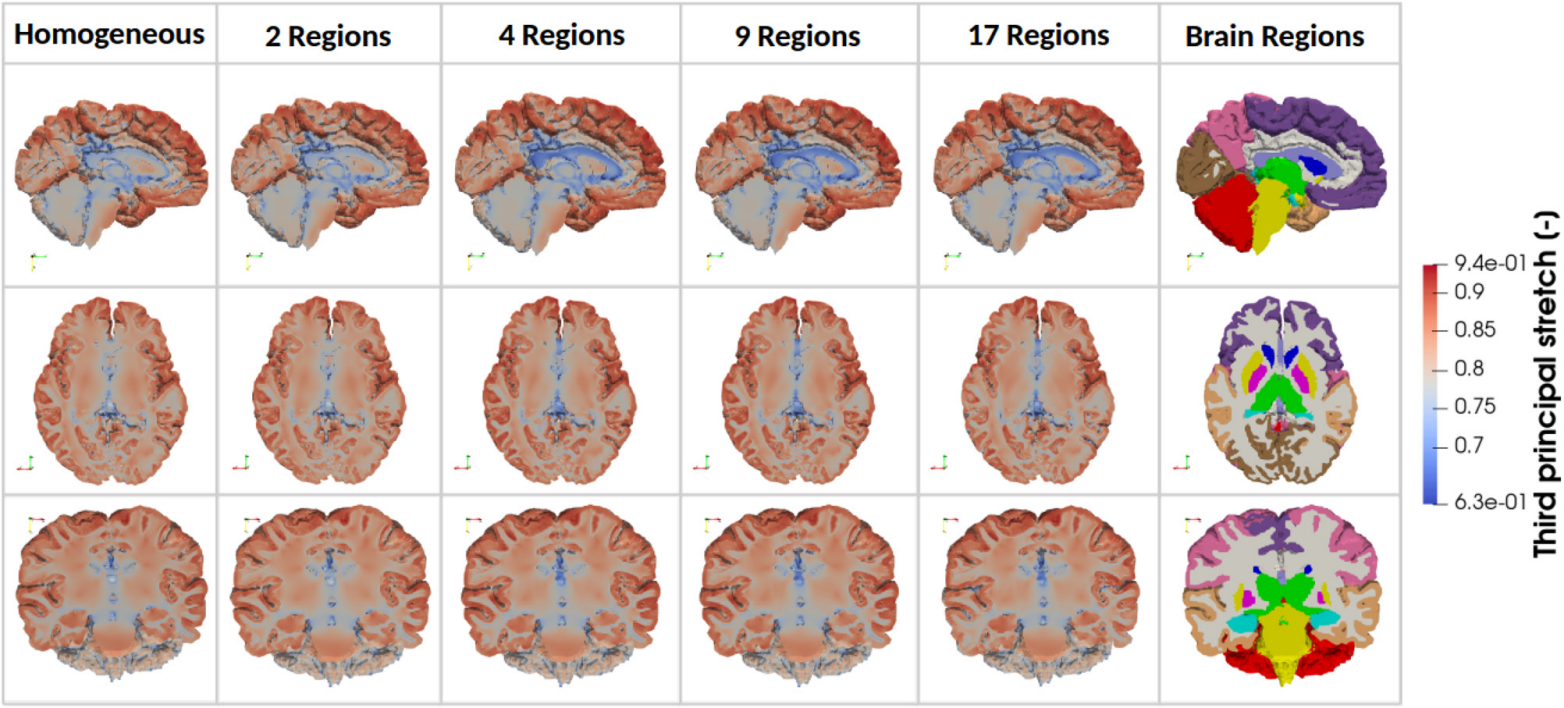
Simulated third principal stretch in three anatomical planes for one representative brain model with increasing regional heterogeneity from left to right (homogeneous, 2 Regions, 4 Regions, 9 Regions, and 17 Regions). The considered brain regions are indicated on the right.

*Third principal stretches*. Figure 8 provides a visualization of the third principal stretch, representing the compressive deformation experienced by brain tissue during atrophy, with lower values indicating greater levels of compression, for one representative brain.

Across all models, the corpus callosum exhibits the lowest third principal stretch values, indicating the highest compressive deformation. This is followed by the internal brain structures and the corona radiata. In contrast, the cortical regions display higher values, suggesting they experience comparatively less compression.

**FIG. 9.**
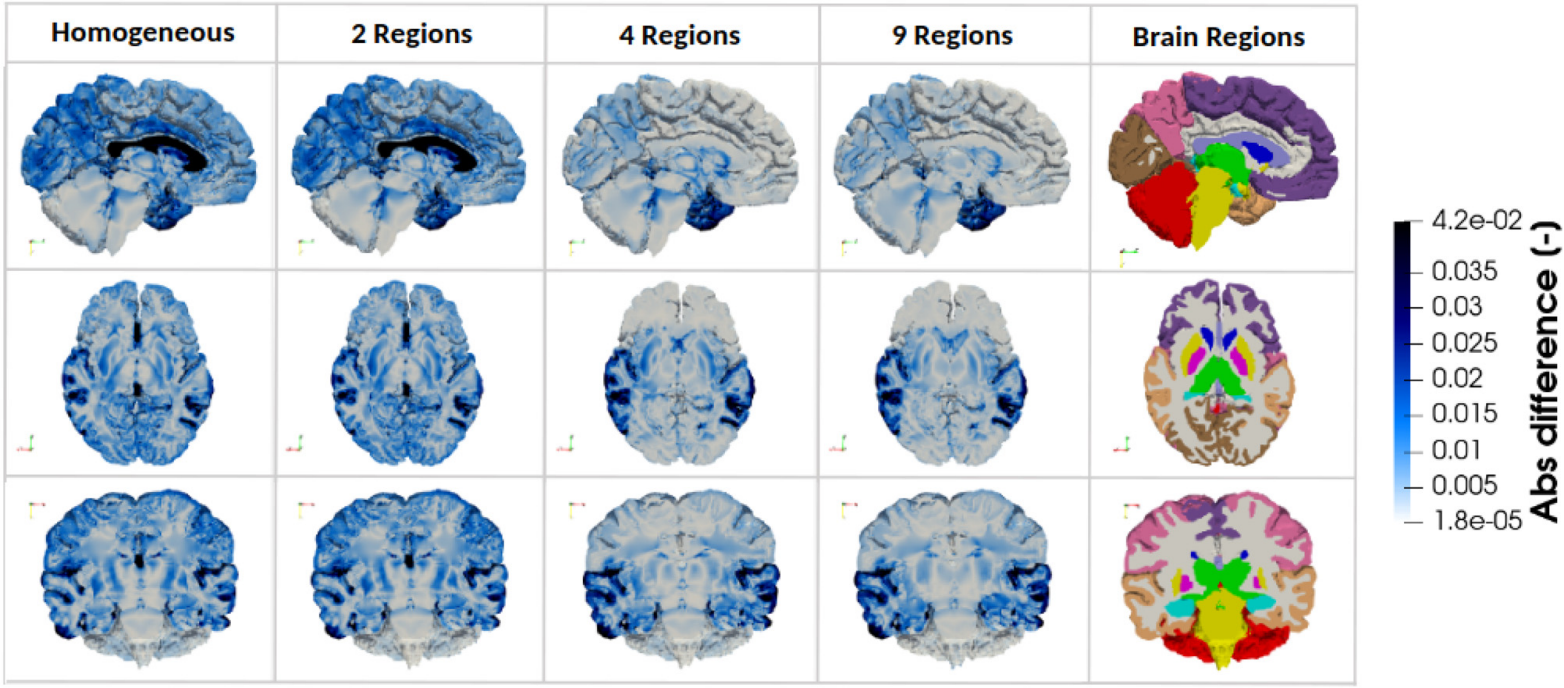
Absolute difference in third principal stretch between the fully heterogeneous model (17 Regions) and the four simplified models (homogeneous material properties, 2 Regions, 4 Regions, and 9 Regions) in three anatomical planes, for one representative 3D brain model.

**FIG. 10.**
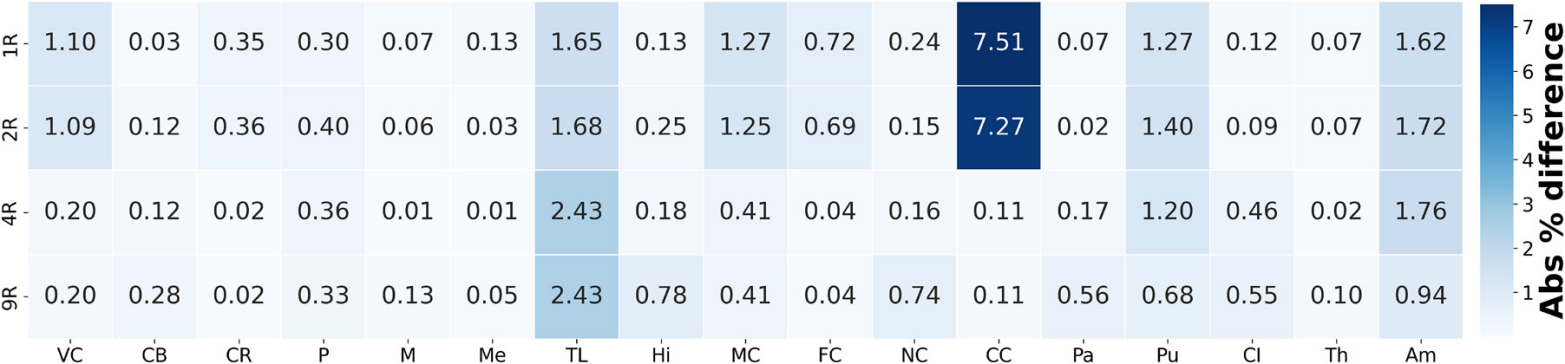
Heatmap of absolute percentage difference in average third principal stretch in each region (averaged over all 3D brain meshes) between the fully heterogeneous model (17 Regions) and the four simplified regional models (homogeneous material properties, 2 Regions, 4 Regions, 9 Regions, and 17 Regions).

Figure 9 illustrates the absolute differences in third principal stretch between each simplified regional model and the fully heterogeneous 17R model, for one representative brain. Additionally, Fig. 10 provides a quantitative summary of these differences, visualizing the absolute percentage changes across brain regions, averaged over the 6 3D brains.

The most pronounced discrepancies are observed in the corpus callosum, especially for the 1R and 2R models, which deviate significantly from the fully detailed 17R model. In contrast, the 4R and 9R models provide closer approximations to the 17R. Additional differences are noted in the temporal lobe, where the absolute percentage difference in the average third principal stretch increases for the 4R and 9R models, although these remain relatively small, approximately 3%. Other regional differences, seen in the amygdala and motor cortex, are also apparent in Fig. 9. While the 1R and 2R models clearly deviate from the 17R model, the degree of deviation for the 4R and 9R models is less clear. Therefore, a statistical analysis focusing on the comparison between the 9R and 17R models was performed to better evaluate the significance of these differences.

#### 4. Statistical analysis

Unlike hydrostatic stress, where regional differences are evident (Figs. 5–7), the principal stretches exhibit more subtle regional sensitivity, with less pronounced differences observed between the 9R and 17R models. To evaluate the significance of these differences, a statistical analysis was performed.

**FIG. 11.**
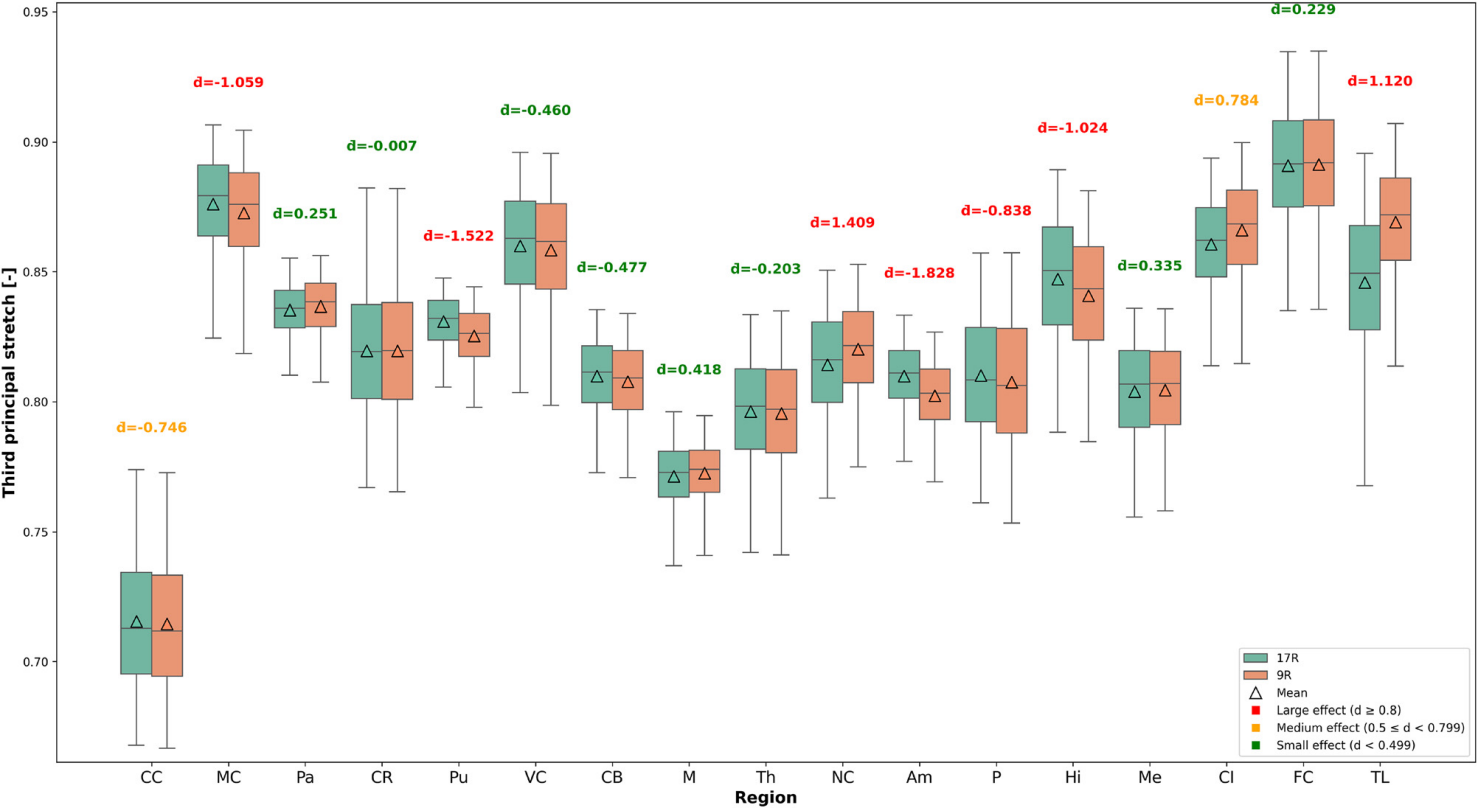
Box plots comparing third principal stretch distributions between 17R and 9R models across brain regions. Box plots display median (centerline), mean (triangle), interquartile range (box), and whiskers extending to the 5th and 95th percentiles for enhanced visual clarity. Cohen's d values are displayed above each region to indicate effect size magnitude.

Figure 11 displays box plots comparing the third principal stretch distributions between the 17R and 9R models for all brain regions, across the analyzed 3D brains. Each plot includes the median (centerline), mean (triangle), interquartile range (box), and whiskers spanning the 5th to 95th percentiles. The Wilcoxon signed-rank test yielded p-values <0.001 for all regions, indicating statistically significant differences between the two models. However, due to the large sample size, such non-parametric tests can become highly responsive to even minimal differences in distributions. Therefore, Cohen's d was also calculated, for each brain and each region to assess practical significance. The mean Cohen's d value across all 6 3D brains was computed for each region, and the values are annotated above each region in the boxplot.

The results indicate that the pallidum, corona radiata, visual cortex, cerebellum, midbrain, thalamus, medulla, and frontal cortex exhibited small effect sizes, suggesting that the actual magnitude of difference in these regions between the two models is relatively minor. The corpus callosum and cortex insula showed medium effect sizes, implying moderate practical relevance. In contrast, several regions, including the motor cortex, putamen, nucleus caudatus, amygdala, pons, hippocampus, and temporal lobe, displayed large effect sizes, reflecting significant differences between the models.

These results indicate that even small absolute percentage differences (as shown in Fig. 10) can be meaningful when considering the underlying data distribution, sample size, and standard deviation, and reinforce the importance of using the more detailed 17R model over the 9R model.

Box plots for the remaining output measures, including other stress and stretch components, are provided in Appendix C.

### B. Effects of atrophy on morphology

Our results regarding the effect of regional heterogeneity on the brain's mechanical response to atrophy have highlighted the significant differences between the 17 Region (17R) model and the less heterogeneous ones. Therefore, we proceed with simulations using the 17R model to study the impact of atrophy on morphological changes in the brain and the evolution of stress with atrophy.

Atrophy simulations were conducted on six individual brain models using the 17R model. These simulations resulted in an overall brain volume reduction ranging from approximately 20% to 25%.

**FIG. 12.**
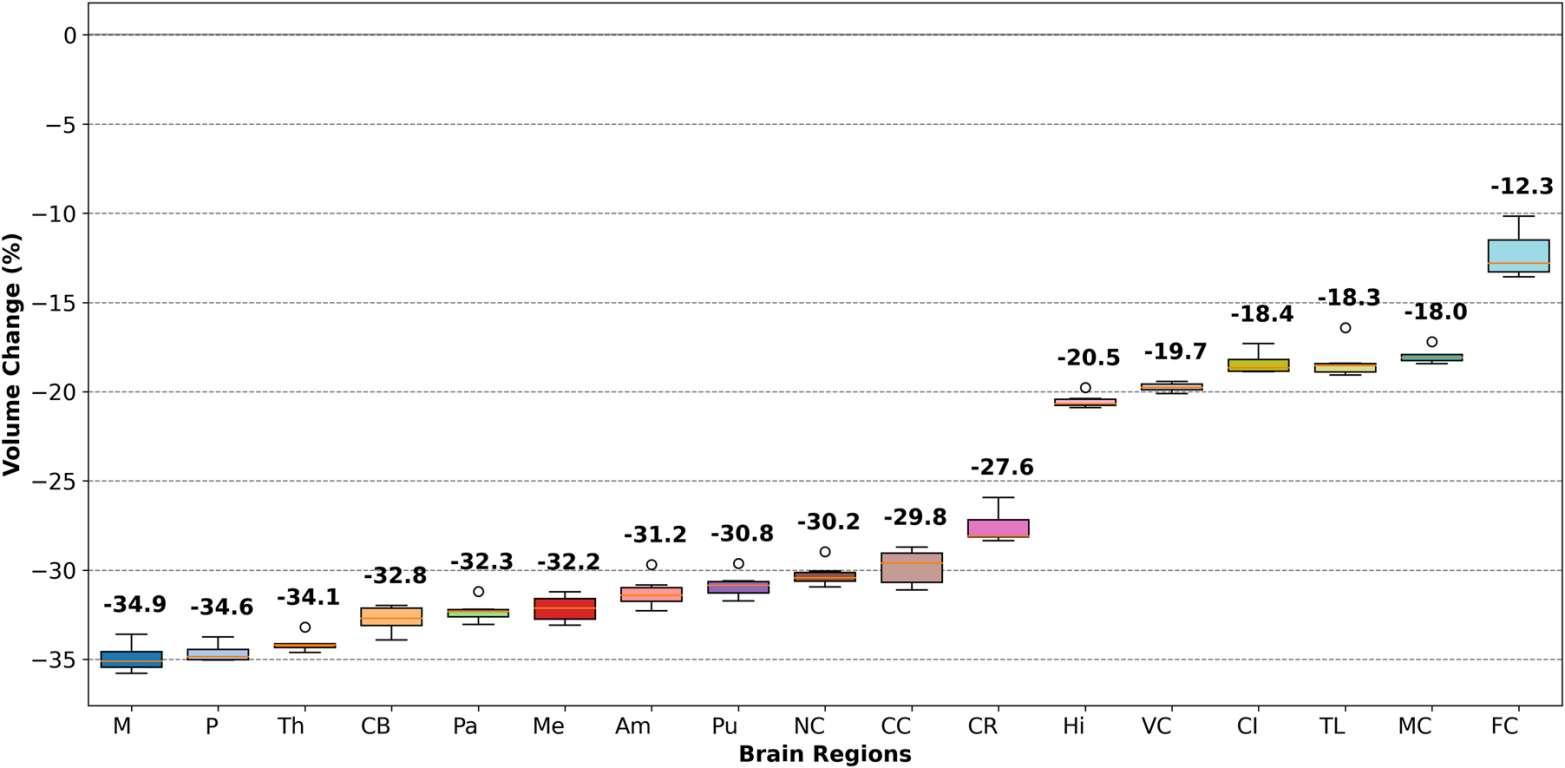
Boxplot illustrating the percentage change in volume for each brain region across all six brains.

Figure 12 presents box plots summarizing the percentage volume change in each brain region across the six models, for all regions. Regional volume reductions range from −12.3% to –34.9% on average. The pattern of atrophy generally aligns with the compressive stretch trends observed in Fig. 8. The cortex shows the smallest volume loss, while the hippocampus exhibits moderate atrophy, with an average reduction of around 20%.

The most pronounced volume loss occurs in the corona radiata, the internal brain structures, and the corpus callosum.

## III. CONCLUSIONS

One of the many objectives in brain biomechanics research is to predict brain behavior in health and disease. From a mechanical perspective, this requires accurate mechanical characterization of the different regions in the brain to ensure realistic computer simulations. In this study, the focus was placed on cerebral atrophy, a condition morphologically characterized by brain shrinkage. This process can occur physiologically with aging or pathologically as a result of neurodegenerative diseases. Previously developed brain shrinkage models for simulating atrophy were adopted and extended. To increase anatomical detail, the brain was segmented into seventeen regions, with distinct material properties assigned to each. The resulting heterogeneous model was then compared to models of decreasing heterogeneity to evaluate the influence of regional mechanical heterogeneity on simulations outputs. Results indicated that regional heterogeneity had a limited effect on overall morphological changes, with the exception of ventricular enlargement and corpus callosum shrinkage, both of which were more sensitive to regional material properties. Further analysis revealed that mechanical heterogeneity significantly affected local displacement within brain regions, highlighting the importance of detailed regional modeling for accurate predictions of brain kinematics. A detailed investigation of hydrostatic stress and third principal stretch distributions was also performed. While the 1R and 2R models exhibited large deviations from the 17R model, the 4R and 9R models more closely approximated the fully heterogeneous case. Nonetheless, statistical analysis confirmed that significant differences remained in several regions, supporting the use of the 17R model for more accurate mechanical representation. Moreover, our results co-localize with regions known to exhibit selective atrophy and mechanical sensitivity, with important effects in the hippocampus, amygdala, temporal and insular cortices, basal ganglia, corpus callosum, and corona radiata. Persistent differences between the 9R and 17R models in the temporal and insular regions, both sensitive to cerebral atrophy, further underscore the importance of regional differentiation. Despite the insights provided, the study has several limitations. First, although the atrophy model was based on previous work, it was adapted here such that the neurotoxic protein concentration varied spatially across the brain but was assumed to be constant in time. This simplification reduced computational complexity but does not fully reflect the progressive nature of pathological atrophy. Second, the reported stress values were interpreted purely from a mechanical perspective. Further investigation is required to connect these mechanical insights with physiological and clinical relevance. Third, while we explicitly modeled regional heterogeneity, we assumed isotropy within each region. Evidence suggests that the corpus callosum shows direction-dependent trends. Future work could therefore include transversely isotropic constitutive laws in this particular region, contingent on the availability of reliable direction-specific material parameters. Finally, although the 17R model offers a high level of anatomical and mechanical detail, future studies are necessary to validate its predictions against *in vivo* observations.

## IV. METHODS

### A. Heterogeneous brain model

In order to investigate the effects of regional heterogeneity on brain atrophy, we considered a 17 region brain model. These regions were created based on the findings of Ref. 29, where nineteen anatomical regions were tested and then grouped together based on the calculated material parameters and location.

The heterogeneous 17R model was compared against progressively more homogeneous models. The **1R** (homogeneous) model serves as a baseline to quantify the effect of introducing heterogeneity. The **2R** model differentiates between gray and white matter. The **4R** model introduces differences in material parameters between the cortex, corona radiata, corpus callosum, and internal brain structures, motivated by reported parameter contrasts among these regions.^29,30^ The **9R** model extends the 4R by further distinguishing internal brain structures, where prior work found non-negligible discrepancies between 4R and 9R.^27^ Finally, the **17R** (heterogeneous) model adds cortical segmentation and further subdivides internal brain structures. A summary of the regional segmentations is provided in Table II.

We used six three-dimensional brain models created from magnetic resonance imaging (MRI) data in the OASIS (Open Access Series of Imaging Studies) cross-sectional dataset.^31^ All scans used the MPRAGE sequence with in-plane resolution $1.0\times 1.0\;{\text{mm}}^{2}$ and 1.25 mm slice thickness. The demographic information of the selected subjects is summarized in Table I. These images were preprocessed and segmented using the open-source FreeSurfer image analysis suite (https://surfer.nmr.mgh.harvard.edu/). Additional segmentation of the brainstem was also performed.^32^ An in-house Python script (available at https://github.com/BRAINIACS-Group/Brain-Creation-Code) then generated a hexahedral mesh with $1\times 1\times 1\;{\text{mm}}^{3}$ elements, applied Laplacian surface smoothing at all region boundaries (including inter-regional interfaces and the outer brain surface), and extracted region-wise data.

**TABLE I.** Demographic information for the six subjects used in the study.

Subject	Sex	Age
Brain 1	Female	55
Brain 2	Male	28
Brain 3	Male	18
Brain 4	Female	24
Brain 5	Male	21
Brain 6	Female	20

Consistent with reported subarachnoid CSF thicknesses of approximately 1– 5 mm^33^ and its increase with aging,^34^ we added a 3 mm cerebrospinal fluid (CSF) layer to the outer brain surface. The CSF was meshed with the brain and modeled as an ultrasoft, compressible solid with a stiffness 10 times lower than cortex, following the approach of.^19^ CSF stresses remained minimal relative to brain tissue, and no artificial stress concentrations were observed within the layer.

For mesh sensitivity, we compared the “fine” mesh ( $1\times 1\times 1\;{\text{mm}}^{3}$, comparable to the MRI voxel size) with a “coarse” mesh ( $2\times 2\times 2\;{\text{mm}}^{3}$). Both produced similar displacement/stress fields and consistent regional metrics. We report results with the 1 mm mesh because it better resolves curved interfaces. Across subjects, the resulting meshes contained $1.31–1.9210^{6}$ hexahedral elements. All simulations employed full Gauss integration.

**FIG. 13.**
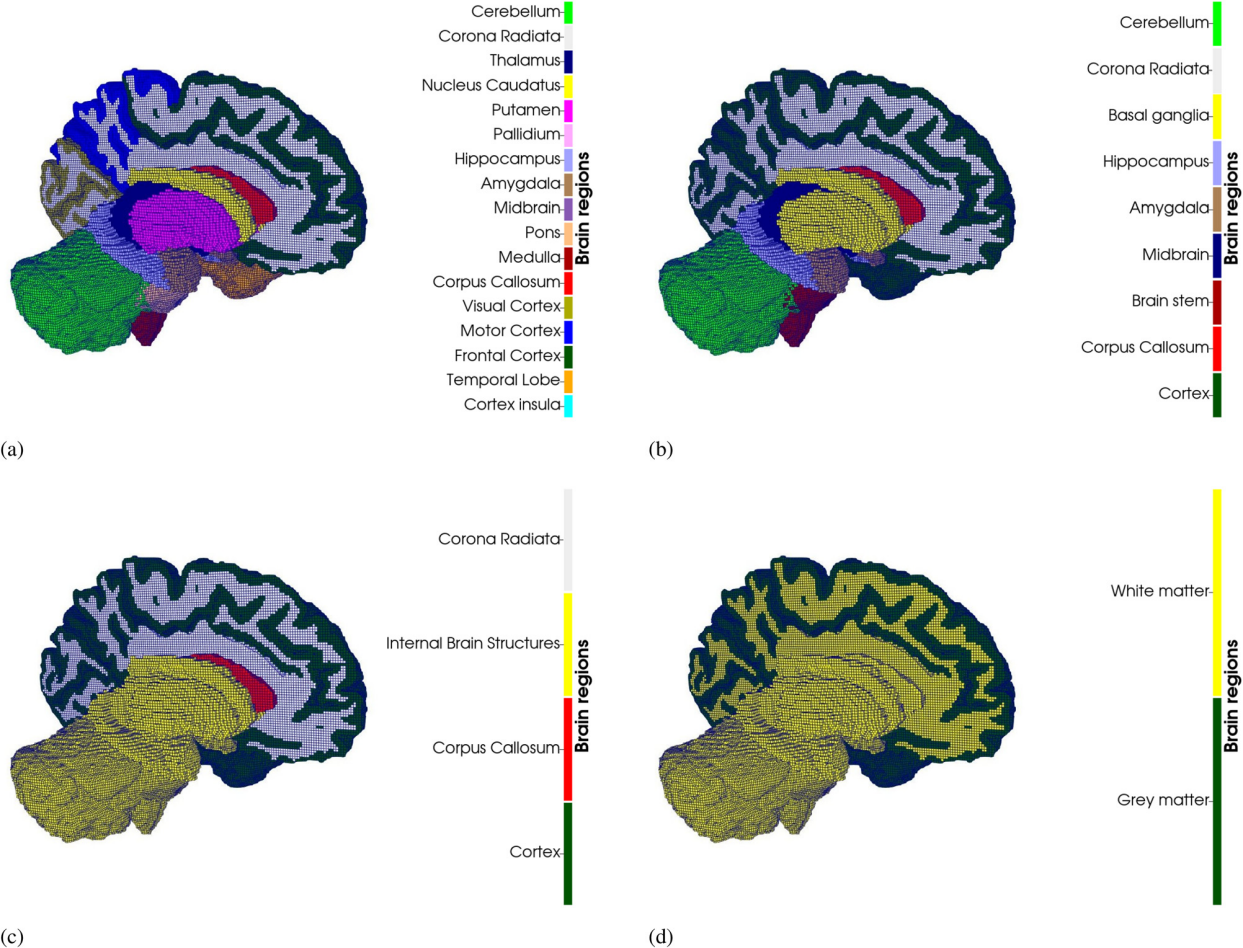
Regional delineation for (a) the 17 Regions (17R) model, (b) the 9 Regions (9R) model, (c) the 4 Regions (4R) model, and (d) the 2 Regions (2R) model. Not included in this figure is the 1 Region (1R) fully homogeneous model. The brains are surrounded by a layer of cerebrospinal fluid (CSF) in the computational model. The CSF is omitted from this schematic for visual clarity (see Fig. 15 for an illustration including the CSF layer).

The brain was segmented into 17 regions: frontal cortex (FC), motor cortex (MC), visual cortex (VC), insular cortex (CI), temporal lobe (TL), corona radiata (CR), nucleus caudatus (NC), pallidum (Pa), putamen (Pu), medulla (Me), pons (P), midbrain (M), thalamus (Th), cerebellum (CB), amygdala (Am), hippocampus (Hi), and corpus callosum (CC) [see Fig. 13(a)].

For each of the six brain models, four comparative models of decreasing heterogeneity [9 Regions (9R), 4 Regions (4R), 2 Regions (2R), and 1 Region (1R)] were also created to compare with the 17 Regions (17R) model, as shown in Fig. 13. The segmentation details of each model are given in Table II.

**TABLE II.** Segmentation of five brain models based on decreasing heterogeneity.

17 Regions	9 Regions	4 Regions	2 Regions	1 Region
Frontal cortex	Cortex	Cortex	Cortex	Homogeneous
Motor cortex				
Visual cortex				
Insular cortex				
Temporal lobe				
Corona radiata	Corona radiata	Corona radiata	Subcortical region	
Nucleus caudatus	Basal ganglia	Internal brain structures		
Pallidum				
Putamen				
Medulla	Brain stem			
Pons				
Midbrain	Midbrain			
Thalamus				
Cerebellum	Cerebellum			
Amygdala	Amygdala			
Hippocampus	Hippocampus			
Corpus callosum	Corpus callosum	Corpus callosum		

### B. Constitutive model and material parameters

Since brain atrophy evolves over years, we neglect viscous effects. While several *in vivo* magnetic resonance elastography (MRE) studies have indicated that brain tissue exhibits direction-dependent behavior, particularly in the while matter, and that the degree of anisotropy varies across regions,^35^ recent large-strain, multi-region experiments only confirm a pronounced direction dependence in the corpus callosum, whereas other structures show no statistically significant directionality, implying that anisotropy is not uniform across brain white matter regions.^36^ Following the conclusions in Ref. 36, we model brain tissue as mechanically isotropic at the organ scale but capture regional heterogeneity (via the 1R–17R model models). This choice stems from the current availability of isotropic region-specific material parameters, for all considered brain regions. Accordingly, we employ an isotropic one-term Ogden model, which accurately represents the time-independent hyperelastic response of brain tissue under diverse loading conditions.^22,30,37^

The corresponding strain energy function is split into an isochoric and a volumetric part,^38^

$$\psi =\psi_{\text{iso}}+\psi_{\text{vol}}.$$ (1)

The isochoric part is defined in terms of the isochoric principal stretches ${\bar{\lambda }}_{a}=J^{-1/3}\lambda_{a}$, where *J* denotes the volume ratio J=detF and F is the deformation gradient, and is given by

$$\psi_{\text{iso}}=2\mu /\alpha^{2}({\bar{\lambda }}_{1}^{\alpha }+{\bar{\lambda }}_{2}^{\alpha }+{\bar{\lambda }}_{3}^{\alpha }-3).$$ (2)

The shear modulus μ and the nonlinearity parameter α are determined by fitting the model to experimental data. The volumetric part is defined as

$$\psi_{\text{vol}}=\kappa \frac{1}{4}(J^{2}-1-2\;\text{ln}\;J),$$ (3)

where κ, the bulk modulus, is determined from the shear modulus and the initial Poisson's ratio, ν, through the relation

$$\kappa =\mu \frac{2(1+\nu )}{3(1-2\nu )}.$$ (4)

The Cauchy stress tensor σ is derived from the strain energy function ψ through the relation

$$\sigma =J^{-1}\frac{\partial \psi }{\partial F}F^{T}.$$ (5)

Griffiths *et al.*^27^ demonstrated that the choice of Poisson's ratio can significantly affect stress distributions in brain simulations, with lower values (e.g., 0.45) leading to stresses over 40% higher in some regions compared to higher values (e.g., 0.49). This highlights the critical importance of selecting an appropriate Poisson's ratio. In this study, we chose a Poisson ratio of 0.49 and preconditioned material. The material parameters of each region were derived from Ref. 29 and can be found in Table IV in the Appendix A. The material parameters of the 9R, 4R, 2R, and 1R models were calculated using the volume average of the 19R parameters. Only 17 regions were used here instead of 19, as the cerebellar white matter and cerebellar nucleus were grouped as the cerebellum, and the corona radiata and white matter results were similarly collected together as the corona radiata (see Fig. 13).

### C. Modeling cerebral atrophy

Our cerebral atrophy model is based on the work of Budday and Kuhl,^23^ where cerebral atrophy is modeled as volume shrinkage, and the work of Blinkouskaya *et al.*,^19^ where both natural and pathological atrophy are considered. In diseases such as Alzheimer's, atrophy is suggested to be accelerated due to the spreading and accumulation of misfolded, neurotoxic proteins. As we are only interested in how mechanical differences between the various regions of the brain affect the results, we have not modeled the diffusion of these proteins, but have rather specified an initial concentration profile on each brain.

To model the volumetric shrinkage of the brain, a classical multiplicative split of the deformation gradient into an elastic part $F^{e}$ and an atrophy part $F^{a}$ is used such that

$$F=F^{e}\cdot F^{a}\;\text{and}\;J=\text{det}(F)=J^{e}J^{a}.$$ (6)

The multiplicative split here extends to the Jacobian *J*, where $J^{e}$ is the elastic volume change and $J^{a}$ is the volume loss due to atrophy.

The mechanical behavior is now characterized as an atrophy-weighted strain energy function such that

$$\psi_{0}=J^{a}\psi .$$ (7)

Following standard arguments of thermodynamics, the Cauchy stress from Eq. (5) is thus parameterized exclusively in terms of the elastic tensor $F^{e}$ and its Jacobian $J^{e}$.^17^

Assuming isotropic volume shrinkage, we define

$$F^{a}=\sqrt[{3}]{\theta }I\;\text{and}\;F^{e}=\frac{F}{\sqrt[{3}]{\theta }I},$$ (8)

where $\theta =J^{a}$ is a measurement of atrophy or volume loss.

Following the work of Blinkouskaya *et al.*,^19^ the evolution of the amount of atrophy is differentiated between healthy and accelerated aging. The brain is assumed to shrink at a natural rate of $G_{h}$, while the presence of a high concentration of misfolded proteins, *c*, results in an accelerated rate, $G_{c}$. The evolution equation is formulated such that if the concentration is above a threshold, $c^{\text{crit}}$, the atrophy is accelerated using a Heaviside step function $H(c-c^{\text{crit}})$ such that

$$\dot{\theta }=[1+\gamma (c)]G_{h}=\{\begin{array}{cc} G_{h} & \text{if}\;c<c^{\text{crit}},\\ G_{h}+G_{c} & \text{if}\;c\geq c^{\text{crit}}, \end{array}$$ (9)

where $\gamma (c)=\frac{G_{c}}{G_{h}}H(c-c^{\text{crit}})$.

To implement progressive shrinkage, we solve the problem in a quasi-static, incremental framework with consecutive time steps. At each increment n→n+1, the atrophy measure $\theta^{\;n+1}$ is updated and mapped into the atrophy tensor $F_{a}^{\;n+1}={\left(\theta^{\;n+1}\right)}^{1/3}I$. The elastic equilibrium is then computed for $F_{e}^{\;n+1}$ with $F=F_{e}F_{a}$. Because the physiological timescale of atrophy (months–years) is much longer than elastic relaxation times, we neglect viscosity and treat each increment as a quasi-static equilibrium step.

As the pattern of atrophy is not homogeneous throughout the brain, we set differing atrophy rates in the white matter and the gray matter. The parameters follow the approach in Ref. 19 (Table III) and are reported in % per unit time. Negative values denote shrinkage. Consistent with prior observations, white matter atrophy is assumed greater than gray matter atrophy.^6,39^

**TABLE III.** Atrophy parameters with healthy and pathological atrophy rates in gray matter, white matter, and the hippocampus.

	G_*h*_ (%/year)	G_*c*_ (%/year)
Gray matter	−0.1	−0.2
Hippocampus	−0.1	−0.2
White matter	−0.15	−0.35

**FIG. 14.**
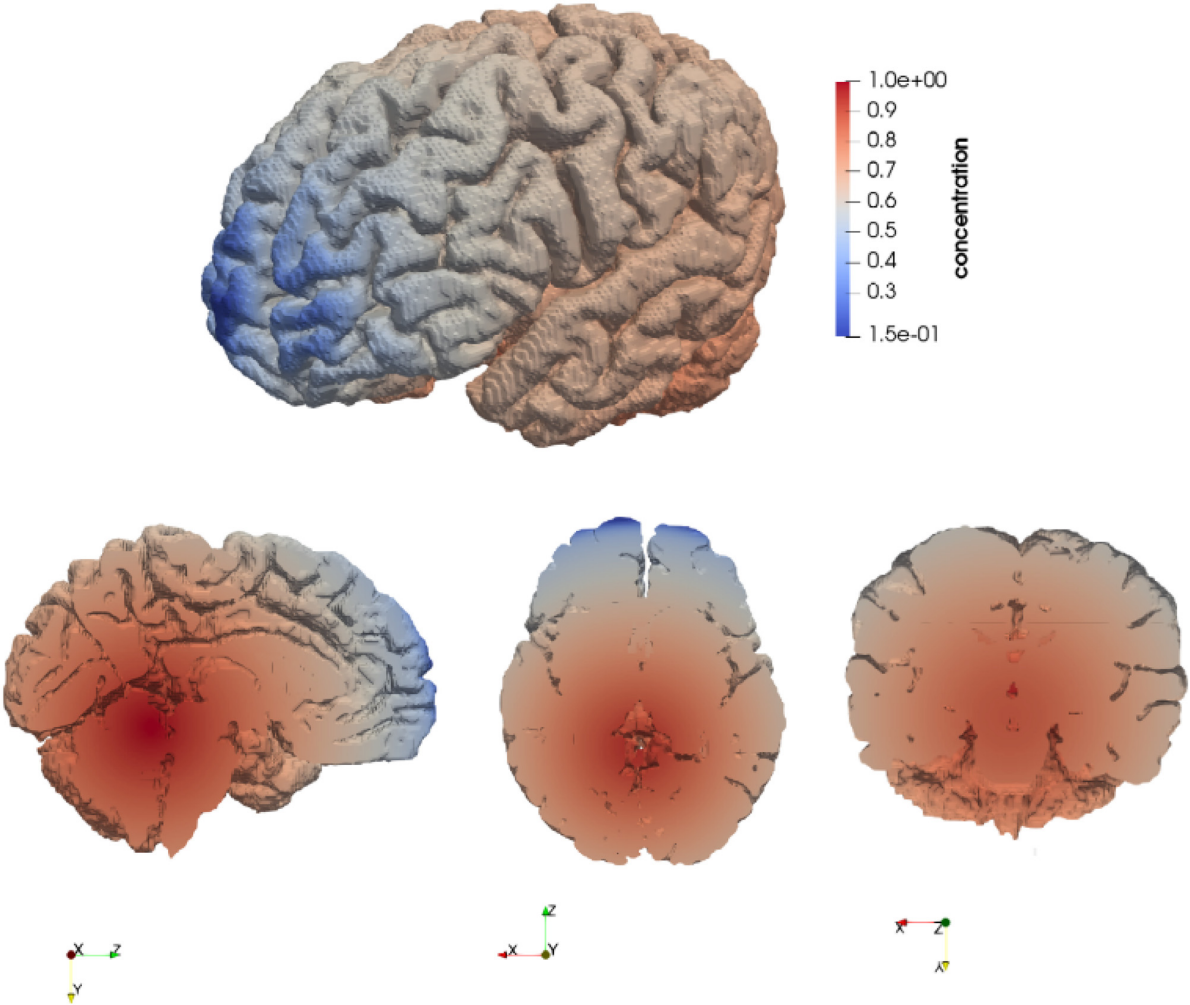
Concentration profile of the misfolded proteins. The concentration is set to be maximal at the center, defined relatively to the brainstem and the hippocampus, and decreases linearly with distance from the center.

**FIG. 16.**
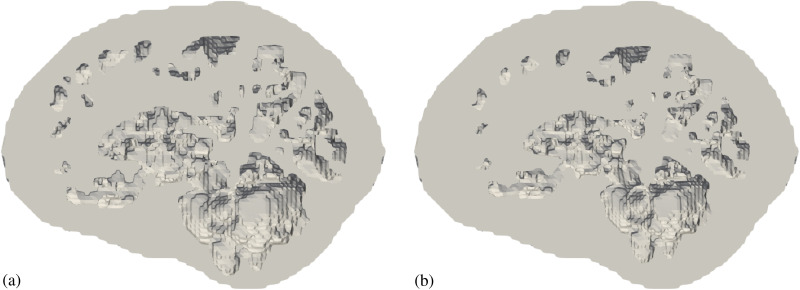
Sagittal cross sections before (a) and after (b) atrophy. The brain is clipped to show only the CSF layer, which appears thicker after atrophy, corresponding to an approximate 30% increase in CSF volume.

**FIG. 17.**
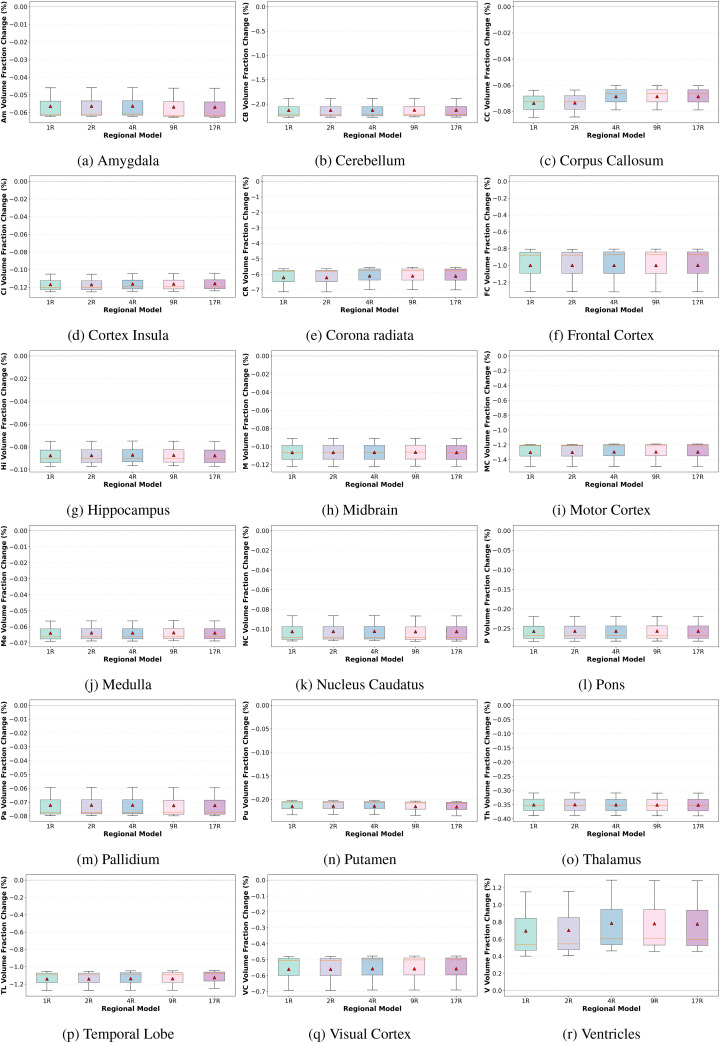
Boxplots showing the change in volume fraction across the simulated brains for each regional segmentation across all 17 Regions and the ventricles. Volume fraction change was quantified as the difference between the initial volume fraction (prior to atrophy) and the final volume fraction at the end of the simulation. Subplots correspond to: (a) amygdala, (b) cerebellum, (c) corpus callosum, (d) cortex insula, (e) corona radiata, (f) frontal cortex, (g) hippocampus, (h) midbrain, (i) motor cortex, (j) medulla, (k) nucleus caudatus, (l) pons, (m) pallidum, (n) putamen, (o) thalamus, (p) temporal lobe, (q) visual cortex, and (r) ventricles.

**FIG. 18.**
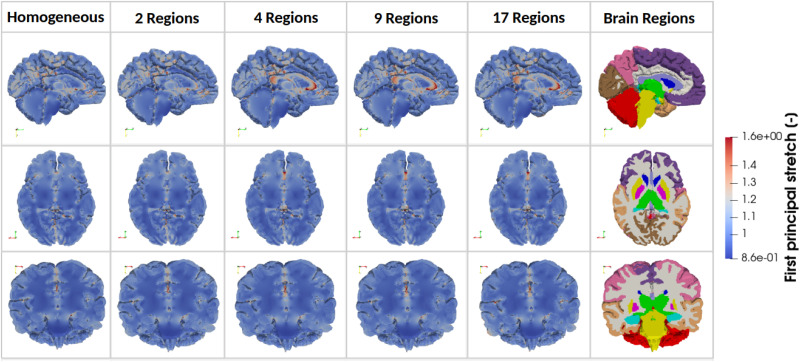
Simulated first principal stretch in three anatomical planes for brain models with increasing regional heterogeneity from left to right (homogeneous, 2 Regions, 4 Regions, 9 Regions, and 17 Regions). The considered brain regions are indicated on the right.

**FIG. 19.**
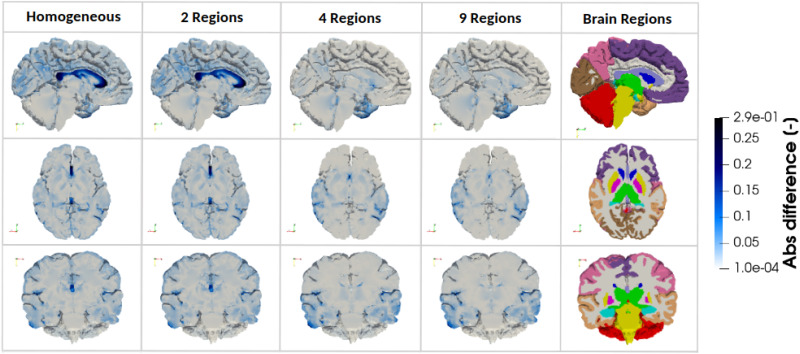
Absolute difference in simulated first principal stretch between the fully heterogeneous model (17 Regions) and the four simplified models (homogeneous material properties, 2 Regions, 4 Regions, and 9 Regions) in three anatomical planes.

**FIG. 20.**
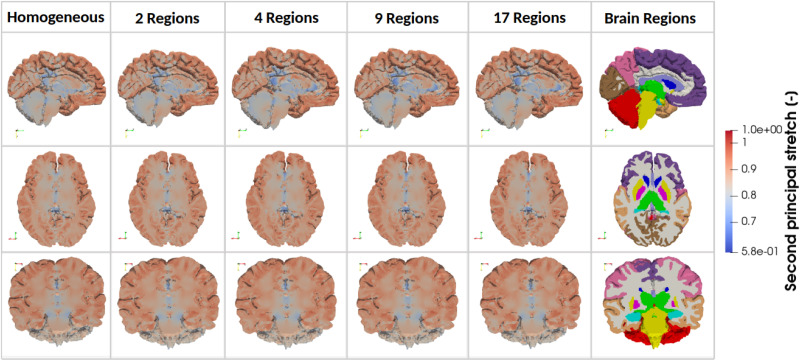
Simulated second principal stretch in three anatomical planes for brain models with increasing regional heterogeneity from left to right (homogeneous, 2 Regions, 4 Regions, 9 Regions, and 17 Regions). The considered brain regions are indicated on the right.

**FIG. 21.**
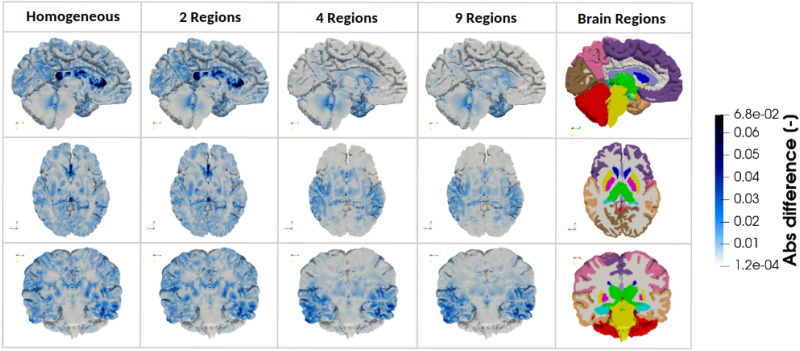
Absolute difference in simulated second principal stretch between the fully heterogeneous model (17 Regions) and the four simplified models (homogeneous material properties, 2 Regions, 4 Regions, and 9 Regions) in three anatomical planes.

**FIG. 22.**
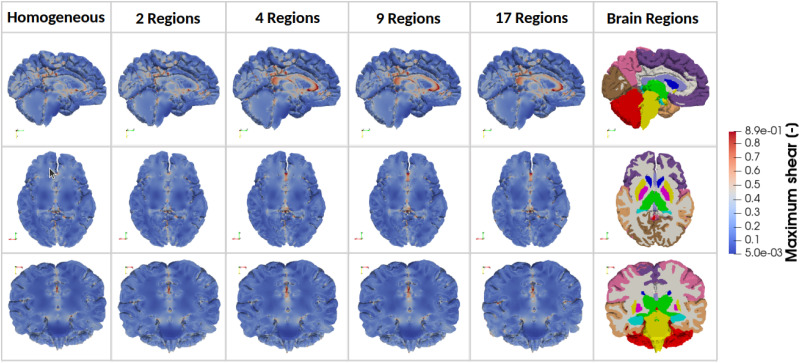
Computed maximum shear in three anatomical planes for brain models with increasing regional heterogeneity from left to right (homogeneous, 2 Regions, 4 Regions, 9 Regions, and 17 Regions). The considered brain regions are indicated on the right.

**FIG. 23.**
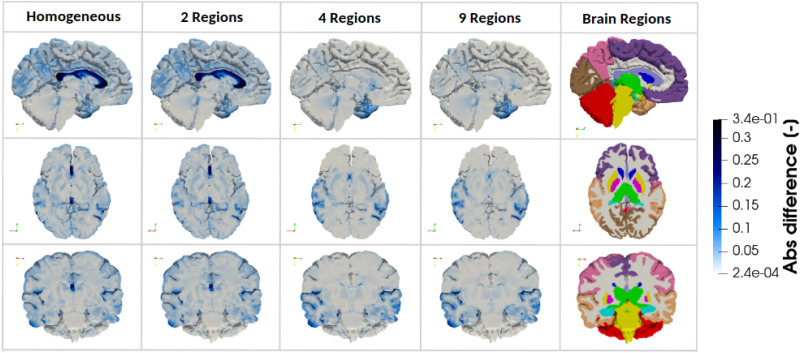
Absolute difference in simulated maximum shear between the fully heterogeneous model (17 Regions) and the four simplified models (homogeneous material properties, 2 Regions, 4 Regions, and 9 Regions) in three anatomical planes.

**FIG. 24.**
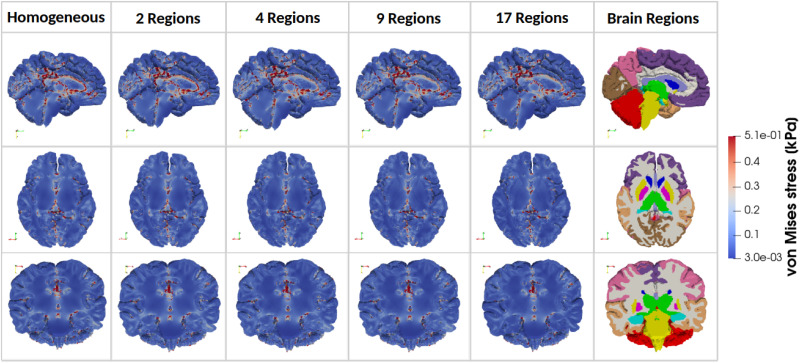
Simulated von Mises stresses in three anatomical planes for brain models with increasing regional heterogeneity from left to right (homogeneous, 2 Regions, 4 Regions, 9 Regions, and 17 Regions). The considered brain regions are indicated on the right.

**FIG. 25.**
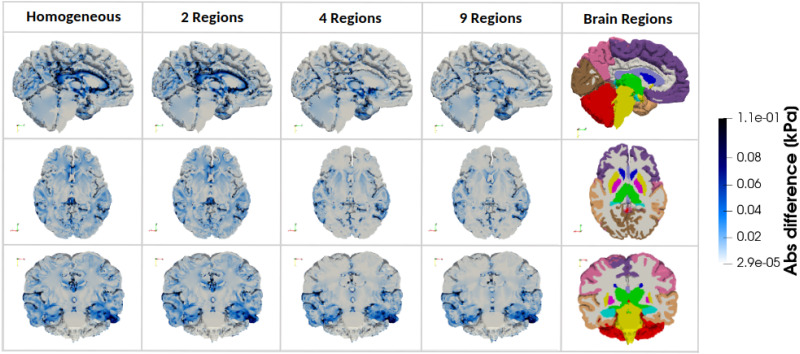
Absolute difference in simulated von Mises stress between the fully heterogeneous model (17 Regions) and the four simplified models (homogeneous material properties, 2 Regions, 4 Regions, and 9 Regions) in three anatomical planes.

The spatial misfolded protein concentration profile, shown in Fig. 14, was designed to approximate the distribution reported in Ref. 19 at an advanced stage of atrophy. A synthetic concentration field was defined, with its center determined anatomically: the x-coordinate was set to the centroid of the brainstem, the y-coordinate to the centroid of the hippocampus, and the z-coordinate to the superior extent of the hippocampal bounding box. The radius of the field was defined as the maximum distance from this center to the boundaries of the entire brain mesh. Within this radius, the concentration was prescribed to be 1 at the center and to decrease linearly with distance, reaching zero at the outer boundary.

### D. Boundary conditions

Previous models of brain atrophy in the literature have employed different boundary conditions. For instance, Refs. 14 and 17 applied Dirichlet boundary conditions at the brainstem, fixing it in space. However, most brain regions experience some level of shrinkage, thinning, or enlargement. In Alzheimer's disease, the brainstem itself undergoes atrophy,^40,41^ suggesting that boundary conditions should not be applied directly to the brain. Later models, such as those proposed by Refs. 42 and 43, instead applied a homogeneous Dirichlet boundary conditions at the skull. Since the skull is rigid and does not deform, this approach offers a more physiologically realistic representation of boundary constraints. In our model, we adopt this strategy by enforcing a homogeneous Dirichlet conditions on the outer boundary of the cerebrospinal fluid (CSF) space, corresponding to the inner surface of the skull.

### E. Mechanical readouts

To characterize the tissue response, we evaluated five mechanical readouts: displacement, principal stretches, maximum shear, hydrostatic stress, and von Mises stress. Kinematic quantities are computed from the deformation gradient F and the left Cauchy–Green tensor $b=FF^{⊤}$; stresses are described by the Cauchy stress tensor σ.

#### 1. Displacement field (mm)

The displacement field is defined as u(X,t)=x(X,t)−X, where X denotes the material (reference) coordinates and x(X,t) the spatial position at time *t*. We report its magnitude $u=||u|{|}_{2}$, which quantifies the movement of each point from its initial position.

#### 2. Principal stretches (-)

The principal stretches $\lambda_{i}$ are defined as the square roots of the eigenvalues of b, i.e., $b\;n_{i}=\lambda_{i}^{2}\;n_{i},$ with ( i=1,2,3), and the principal directions $n_{i}$. They quantify extension/compression along the principal directions $n_{i}$ ( $\lambda_{i}>1$ indicates tension; $\lambda_{i}<1$ indicates compression).

#### 3. Maximum shear (-)

The maximum shear characterizes the most severe shear deformation, i.e., the sliding of parallel internal surfaces past one another, in the tissue,

$$\gamma_{\text{max}}=\text{max}\{\lambda_{1},\lambda_{2},\lambda_{3}\}-\text{min}\{\lambda_{1},\lambda_{2},\lambda_{3}\}=\lambda_{1}-\lambda_{3}.$$

#### 4. Hydrostatic stress (kPa)

The hydrostatic mean stress, $\sigma_{\text{hyd}}$, is computed as

$$\sigma_{\text{hyd}}=\frac{1}{3}\;\text{tr}(\sigma ).$$

It represents the pressure-like component of stress that changes volume without shear. In our convention, negative values denote compression.

#### 5. von-Mises stress (kPa)

The von Mises stress, $\sigma_{VM}$, is computed from the deviatoric Cauchy stress $s=\sigma -\frac{1}{3}\text{tr}(\sigma )I$ as

$$\sigma_{\text{VM}}=\sqrt{\frac{3}{2}\;s:s}.$$

It reduces the complex three-dimensional stress state to a single value, highlighting regions experiencing significant distortion during atrophy.

### F. Statistical analysis

**FIG. 26.**
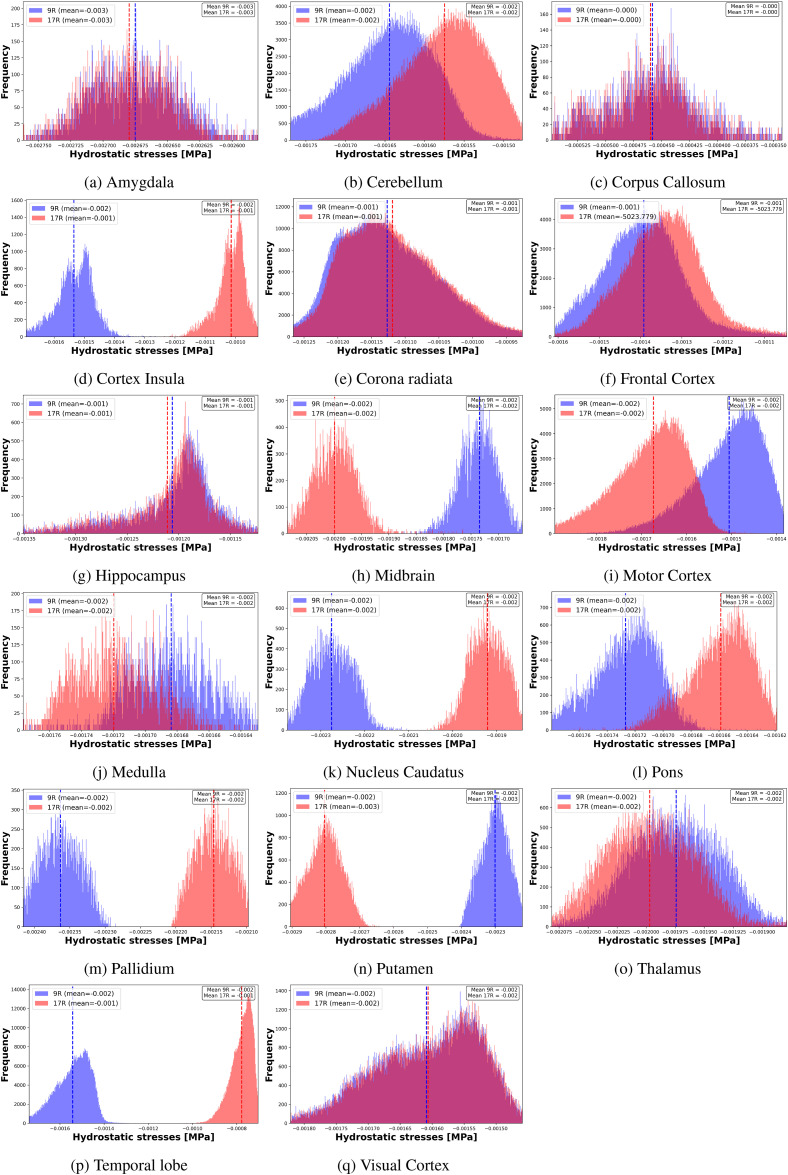
Histograms of the hydrostatic stress distribution across all brain regions for the 9R (blue) and 17R (red) models. The mean values for each model are indicated by dashed lines in their respective colors. Data were cropped to the 1st–99th percentile range prior to plotting to reduce the impact of extreme outliers.

**FIG. 27.**
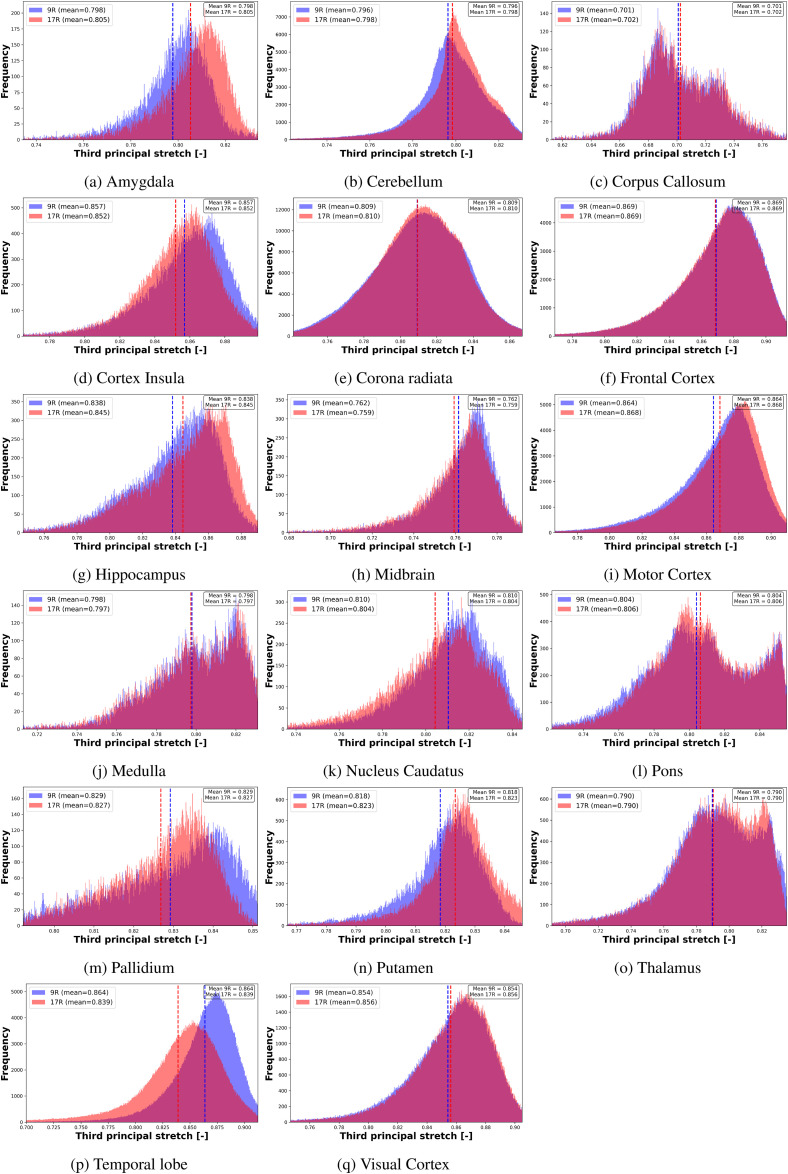
Histograms of the third principal stretch distribution across all brain regions for the 9R (blue) and 17R (red) models. The mean values for each model are indicated by dashed lines in their respective colors. Data were cropped to the 1st–99th percentile range prior to plotting to reduce the impact of extreme outliers.

**FIG. 28.**
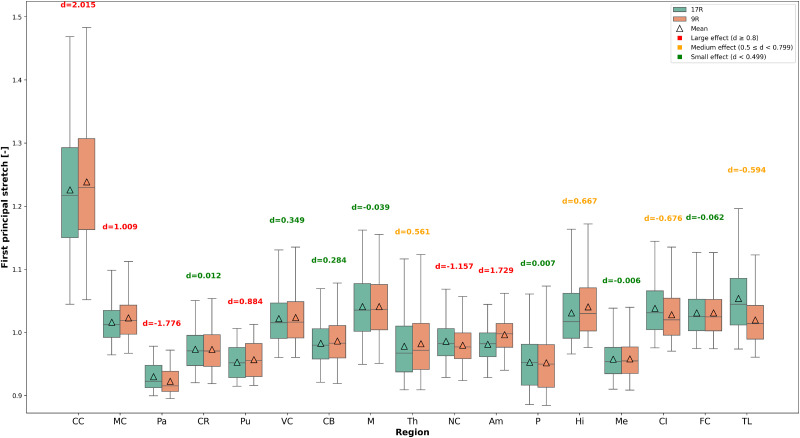
Box plots comparing first principal stretch distributions between 17R and 9R models across brain regions. Box plots display median (centerline), mean (triangle), interquartile range (box), and whiskers extending to the 5th and 95th percentiles for enhanced visual clarity. Cohen's d values are displayed above each region to indicate effect size magnitude.

**FIG. 29.**
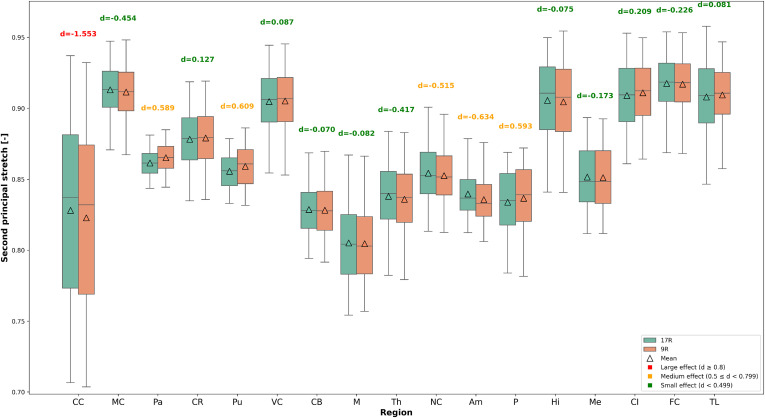
Box plots comparing second principal stretch distributions between 17R and 9R models across brain regions. Box plots display median (centerline), mean (triangle), interquartile range (box), and whiskers extending to the 5th and 95th percentiles for enhanced visual clarity. Cohen's d values are displayed above each region to indicate effect size magnitude.

**FIG. 30.**
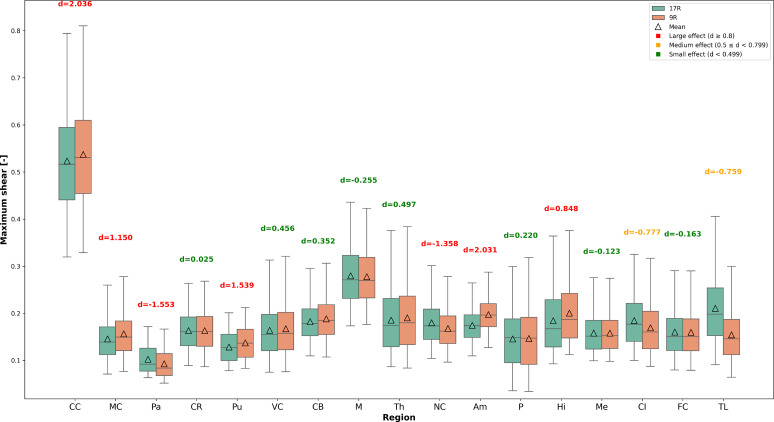
Box plots comparing the maximum shear distributions between 17R and 9R models across brain regions. Box plots display median (centerline), mean (triangle), interquartile range (box), and whiskers extending to the 5th and 95th percentiles for enhanced visual clarity. Cohen's d values are displayed above each region to indicate effect size magnitude.

**FIG. 31.**
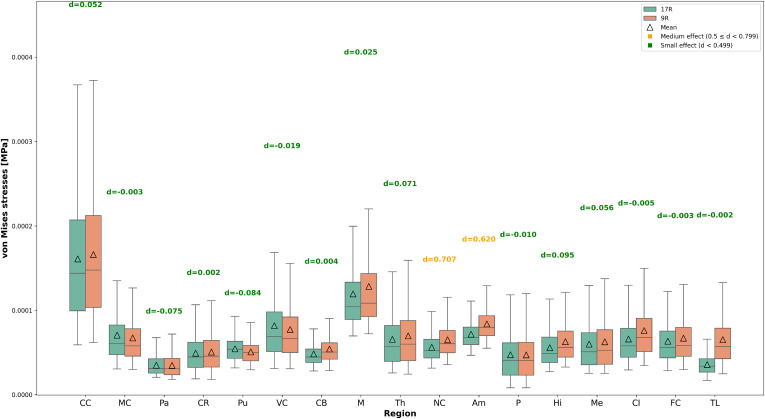
Box plots comparing the von Mises stress distributions between 17R and 9R models across brain regions. Box plots display median (centerline), mean (triangle), interquartile range (box), and whiskers extending to the 5th and 95th percentiles for enhanced visual clarity. Cohen's d values are displayed above each region to indicate effect size magnitude.

**FIG. 32.**
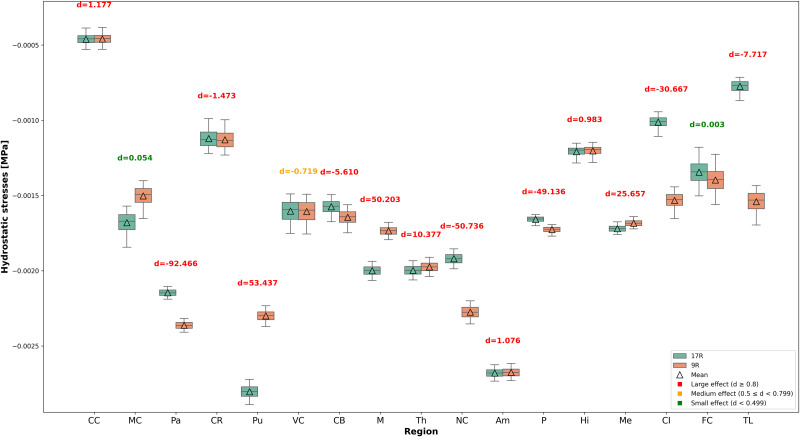
Box plots comparing the hydrostatic stress distributions between 17R and 9R models across brain regions. Box plots display median (centerline), mean (triangle), interquartile range (box), and whiskers extending to the 5th and 95th percentiles for enhanced visual clarity. Cohen's d values are displayed above each region to indicate effect size magnitude.

Atrophy simulations were performed on each brain using the regional segmentation models detailed in Fig. 13. To assess whether the simulation outputs were significantly different between regional models, a statistical analysis was performed. Despite the large number of data points per region, the stress and stretch distributions within the brain were not normally distributed (see Figs. 26 and 27), leading to the use of non-parametric statistical methods. Additionally, since simulations were conducted on the same mesh for each brain with varying heterogeneity, the data were considered paired. As a result, the *Wilcoxon signed-rank test* was employed for analyzing paired non-parametric data. To complement the evaluation, *Cohen's d*, which measures the effect size and determines the practical significance of the calculated p-values, by incorporating both means and standard deviations, was calculated and reported

$$d=\frac{{\bar{x}}_{1}-{\bar{x}}_{2}}{s},$$ (10)

where ${\bar{x}}_{1}$ and ${\bar{x}}_{2}$ are the sample means for each model and *s* is the pooled standard deviation. All statistical analyses were performed using the *scipy* library in *Python*.

## Figures and Tables

**FIG. 15.**
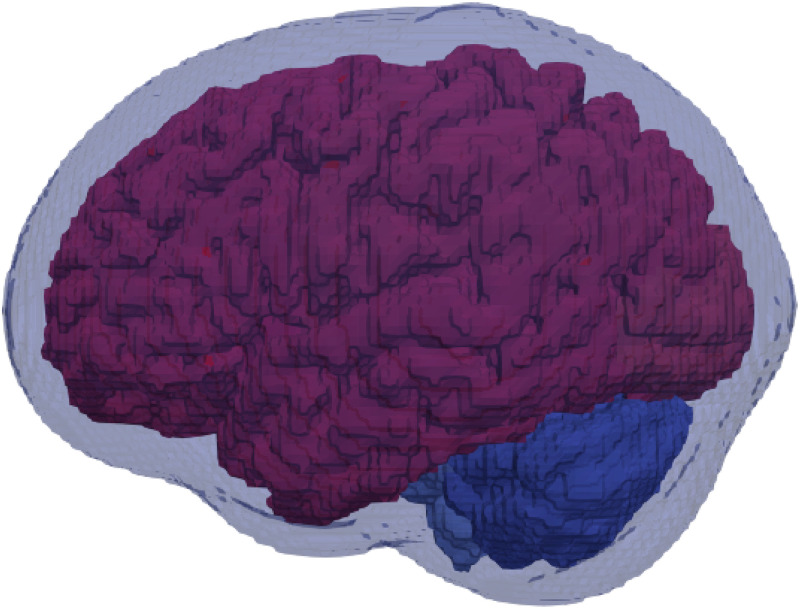
Brain model surrounded by a conforming CSF layer (rendered semi-transparent for visualization).

## Data Availability

Exemplary data generated and analyzed in this study, including corresponding computer code, are available on Zenodo, 2025: doi: 10.5281/zenodo.17979899. Additional data that support the findings of this study are available from the corresponding author upon reasonable request.

## APPENDIX A: MATERIAL PARAMETERS

Material parameters averaged over anatomical regions, extracted from Ref. 29.

**TABLE IV.** Material parameters averaged over anatomical regions, extracted from Ref. 29.

	Preconditioned ν= 45				Preconditioned ν= 49				Unconditioned ν= 45				Unconditioned ν= 49			
	α		μ		α		μ		α		μ		α		μ	
	mean	std.	mean	std.	mean	std.	mean	std.	mean	std.	mean	std.	mean	std.	mean	std.
region																
Am	−19.67	1.80	257.05	116.18	−14.16	1.60	193.34	100.33	−14.74	2.55	386.97	171.00	−10.10	2.26	293.06	133.23
CC	−14.94	12.60	61.66	24.73	−13.72	13.71	37.64	18.26	−12.82	7.88	95.43	34.55	−9.11	6.90	67.82	24.40
CI	−21.20	⋯	205.14	⋯	−19.49	⋯	116.44	⋯	−18.37	⋯	261.34	⋯	−13.99	⋯	188.13	⋯
CR	−19.83	2.96	155.69	67.08	−19.28	5.99	83.33	27.13	−18.65	2.02	209.72	97.64	−15.78	3.89	136.21	57.59
FC	−19.63	0.97	249.69	82.67	−15.90	1.90	168.72	53.82	−15.29	1.73	361.23	127.01	−11.08	1.75	271.89	96.14
Hi	−17.37	2.75	184.21	81.38	−13.09	3.15	129.96	47.59	−13.25	1.58	292.58	147.86	−9.23	1.33	218.74	112.20
M	−18.72	4.87	230.60	99.27	−16.35	6.36	135.61	42.66	−15.07	4.16	338.29	137.77	−11.30	4.13	238.30	91.76
MC	−20.19	1.64	318.98	126.18	−17.99	3.06	195.50	84.99	−16.95	2.06	434.35	168.54	−12.94	2.19	313.65	119.22
Me	−18.90	7.63	228.86	106.41	−17.96	9.30	120.53	41.93	−16.11	7.20	323.46	161.20	−13.15	6.92	216.22	107.02
NC	−15.82	5.85	198.57	99.88	−11.39	5.73	143.46	74.60	−12.58	6.10	277.58	138.29	−8.30	4.94	203.23	96.48
P	−23.23	1.31	241.04	131.47	−22.62	4.91	113.46	48.61	−18.12	3.38	360.56	184.33	−14.43	4.09	241.52	112.86
Pa	−21.16	2.14	242.06	76.14	−17.94	3.75	154.22	60.56	−18.19	3.07	332.32	137.93	−13.67	3.21	240.96	105.92
Pu	−19.24	1.76	305.10	100.76	−15.09	2.90	206.07	55.33	−16.27	2.61	393.89	121.48	−11.86	2.75	285.82	82.46
TL	−8.37	13.00	123.68	23.29	−4.38	11.03	89.51	23.47	−9.18	3.07	202.19	75.27	−5.95	1.97	150.13	61.67
Th	−18.66	5.27	205.38	53.76	−14.32	5.78	139.10	35.73	−15.13	5.63	298.74	89.50	−10.60	4.38	220.34	62.52
VC	−20.81	0.58	299.02	67.23	−19.10	1.09	174.95	47.48	−17.64	1.70	421.88	107.24	−13.62	1.80	306.03	90.17
WM	−19.62	5.66	158.00	82.06	−18.95	8.62	90.34	57.54	−15.81	8.21	224.64	127.98	−12.10	8.11	153.27	89.07
cN	−22.66	4.42	123.64	38.58	−17.43	2.83	89.13	39.37	−17.50	2.53	203.00	84.30	−12.70	2.90	155.49	74.71
cWM	−23.57	7.03	211.15	84.48	−19.65	4.18	131.76	48.53	−20.74	4.43	288.12	101.18	−14.94	3.57	222.22	90.80

## APPENDIX B: ADDITIONAL PLOTS

Brain model surrounded by a conforming CSF layer (rendered semi-transparent for visualization). Sagittal cross sections before (a) and after (b) atrophy. Boxplots showing the change in volume fraction across the simulated brains for each regional segmentation across all 17 Regions and the ventricles.

## APPENDIX C: STATISTICAL PLOTS

Simulated first principal stretch in three anatomical planes for brain models with increasing regional heterogeneity from left to right (homogeneous, 2 Regions, 4 Regions, 9 Regions, and 17 Regions). Absolute difference in simulated first principal stretch between the fully heterogeneous model (17 Regions) and the four simplified models (homogeneous material properties, 2 Regions, 4 Regions, and 9 Regions) in three anatomical planes. Simulated second principal stretch in three anatomical planes for brain models with increasing regional heterogeneity from left to right (homogeneous, 2 Regions, 4 Regions, 9 Regions, and 17 Regions). Absolute difference in simulated second principal stretch between the fully heterogeneous model (17 Regions) and the four simplified models (homogeneous material properties, 2 Regions, 4 Regions, and 9 Regions) in three anatomical planes.Computed maximum shear in three anatomical planes for brain models with increasing regional heterogeneity from left to right (homogeneous, 2 Regions, 4 Regions, 9 Regions, and 17 Regions). Absolute difference in simulated maximum shear between the fully heterogeneous model (17 Regions) and the four simplified models (homogeneous material properties, 2 Regions, 4 Regions, and 9 Regions) in three anatomical planes.Simulated von Mises stresses in three anatomical planes for brain models with increasing regional heterogeneity from left to right (homogeneous, 2 Regions, 4 Regions, 9 Regions, and 17 Regions). Absolute difference in simulated von Mises stress between the fully heterogeneous model (17 Regions) and the four simplified models (homogeneous material properties, 2 Regions, 4 Regions, and 9 Regions) in three anatomical planes. Histograms of the hydrostatic stress distribution across all brain regions for the 9R (blue) and 17R (red) models. The mean values for each model are indicated by dashed lines in their respective colors. Histograms of the third principal stretch distribution across all brain regions for the 9R (blue) and 17R (red) models. The mean values for each model are indicated by dashed lines in their respective colors. Box plots comparing first principal stretch distributions between 17R and 9R models across brain regions. Box plots display median (centerline), mean (triangle), interquartile range (box), and whiskers extending to the 5th and 95th percentiles for enhanced visual clarity. Box plots comparing second principal stretch distributions between 17R and 9R models across brain regions. Box plots display median (centerline), mean (triangle), interquartile range (box), and whiskers extending to the 5th and 95th percentiles for enhanced visual clarity. Box plots comparing the maximum shear distributions between 17R and 9R models across brain regions. Box plots display median (centerline), mean (triangle), interquartile range (box), and whiskers extending to the 5th and 95th percentiles for enhanced visual clarity. Box plots comparing the von Mises stress distributions between 17R and 9R models across brain regions. Box plots display median (centerline), mean (triangle), interquartile range (box), and whiskers extending to the 5th and 95th percentiles for enhanced visual clarity. Box plots comparing the hydrostatic stress distributions between 17R and 9R models across brain regions. Box plots display median (centerline), mean (triangle), interquartile range (box), and whiskers extending to the 5th and 95th percentiles for enhanced visual clarity.
